# The overlooked role of exergames in cognitive-motor neurorehabilitation: a systematic review

**DOI:** 10.1038/s41746-025-01843-4

**Published:** 2025-07-09

**Authors:** Maria Grazia Maggio, Francesca Baglio, Raffaela Maione, Rosalia Calapai, Fulvia Di Iulio, Paulo Cezar Rocha dos Santos, Marcos Maldonado-Díaz, Giulia Pistorino, Antonio Cerasa, Angelo Quartarone, Rocco Salvatore Calabrò

**Affiliations:** 1 IRCCS Centro Neurolesi Bonino-Pulejo, Messina, Italy; 2 IRCCS Fondazione Don Carlo Gnocchi Onlus, Milano, Italy; 3 IRCCS Santa Lucia Foundation, Roma, Italy; 4 IDOR/Pioneer Science Initiative, Rio de Janeiro, Brazil; 5 Physical Medicine and Rehabilitation Service, Clínica Alemana-Universidad del Desarrollo, Santiago, Chile; 6 Università degli studi di Messina, Messina, Italy; 7 Institute of BioImaging and Complex Biological Systems, National Research Council (IBSBC-CNR), Catanzaro, Italy

**Keywords:** Psychology, Signs and symptoms, Neuroscience, Cognitive neuroscience, Motor control

## Abstract

Exergames are emerging tools for cognitive-motor neurorehabilitation, but their effects in adults with neurological conditions remain insufficiently explored. This systematic review examined studies from PubMed, Scopus, Embase, and Web of Science, assessing the impact of exergames on motor and cognitive outcomes. Eligible studies included randomized controlled trials (RCTs), non-RCTs, and experimental designs. The protocol was registered on PROSPERO (CRD420250655053) and followed PRISMA and Cochrane guidelines. Eleven studies (8 RCTs, 2 feasibility studies, 1 secondary analysis) reported improvements in balance, gait, executive function, and memory, with high adherence (85–100%) and minimal adverse effects. While the overall risk of bias was low, heterogeneity in interventions, populations, and outcome measures limited comparability and generalizability. Additionally, the absence of long-term follow-up hindered conclusions on sustained benefits. Exergames appear promising for cognitive-motor rehabilitation in neurological conditions. Future studies should adopt standardized protocols, include long-term follow-up, and explore neurophysiological mechanisms to support clinical implementation.

## Introduction

Optimizing interventions that target rehabilitation for neurological diseases is of utmost importance^1^. Among the interventions, exergames have gained increasing prominence in neurorehabilitation over the past year^2,3^. Exergames, a fusion of “exercise” and “games,” are interactive video games that combine physical activity with digital engagement using motion-sensing technologies to track and respond to players’ movements in real time^4,5^. Exergames are often classified under the broader category of serious games, which refers to digital games designed primarily for purposes beyond entertainment, such as education, training, and health intervention^6–8^. While both exergames and serious games incorporate interactive elements, they differ in their primary mode of engagement. Serious games often focus on cognitive training through gamified neuropsychological tasks and do not necessarily require significant physical movement^6^. In contrast, exergames explicitly integrate physical movement as a core component of gameplay, requiring players to engage in motor activities to interact with the virtual environment^7,9^.

Exergames may incorporate exercise in virtual reality (VR) settings ranging from non-immersive (standard screens with motion sensors) to semi-immersive (projection-based systems) and fully immersive (head-mounted displays with body tracking)^10^. By simulating real-world scenarios in controlled, gamified settings, exergames enable targeted cognitive-motor training. Despite these similarities, exergames and VR-based rehabilitation differ significantly in their approach to movement execution, user agency, and task adaptability. VR rehabilitation encompasses a broad spectrum of applications, ranging from passive observational training to active movement-based interventions that leverage real-time motion tracking. In contrast, exergames specifically prioritize interactive movement as the primary mode of engagement. This distinction is fundamental, as exergames focus on implicit learning, where users enhance their motor control and cognitive flexibility through engaging gameplay mechanics rather than rigid exercise routines^11–13^. Through real-time cognitive engagement, exergames encourage decision-making, problem-solving, and motor adaptation in ecologically valid settings^2,14^. Exergames emphasize an intrinsically motivating experience that encourages long-term adherence and participation. This characteristic makes exergames particularly valuable for individuals with neurological conditions, where conventional rehabilitation methods may struggle to maintain patient adherence and motivation. Given their adaptability, they offer a personalized rehabilitation approach, where clinicians can tailor interventions based on real-time patient feedback and progress monitoring^3^. Several studies suggest that exergames outperform conventional rehabilitation in terms of engagement and long-term adherence, making them a promising tool for neurorehabilitation^15–18^. This aspect is particularly relevant for neurodegenerative and acquired brain disorders, where motor and cognitive impairments frequently coexist. Research has shown that high-intensity coordination training through exergames can lead to functional recovery comparable to regaining years of lost ability, as demonstrated in patients with cerebellar ataxia^19^. Furthermore, exergames have proven to enhance balance and postural stability in Parkinson’s disease and reduce chronic pain and depression in individuals with long-term disabilities^2,14,20,21^. However, existing studies often lack standardized outcome measures, making it difficult to establish clear guidelines for clinical implementation. Therefore, systematically reviewing the literature is timely to understand the current state-of-the-art regarding exergame interference with cognitive-motor outcomes and support optimizing exergame protocols targeting neurorehabilitation.

This review sought to bridge this gap by systematically assessing the impact of exergames on cognitive and motor outcomes. It specifically focuses on individuals with neurological and neurodegenerative disorders, evaluating the effects of exergame-based interventions (compared to conventional rehabilitation or no intervention) on both motor and cognitive functions within a neurorehabilitation context. Thus, it aims to provide critical insights into the potential of exergames as a structured and adaptable rehabilitation approach, with the goal of identifying condition-specific protocols and future directions for optimizing their clinical implementation.

## Results

### Study selection

**Fig. 1:**
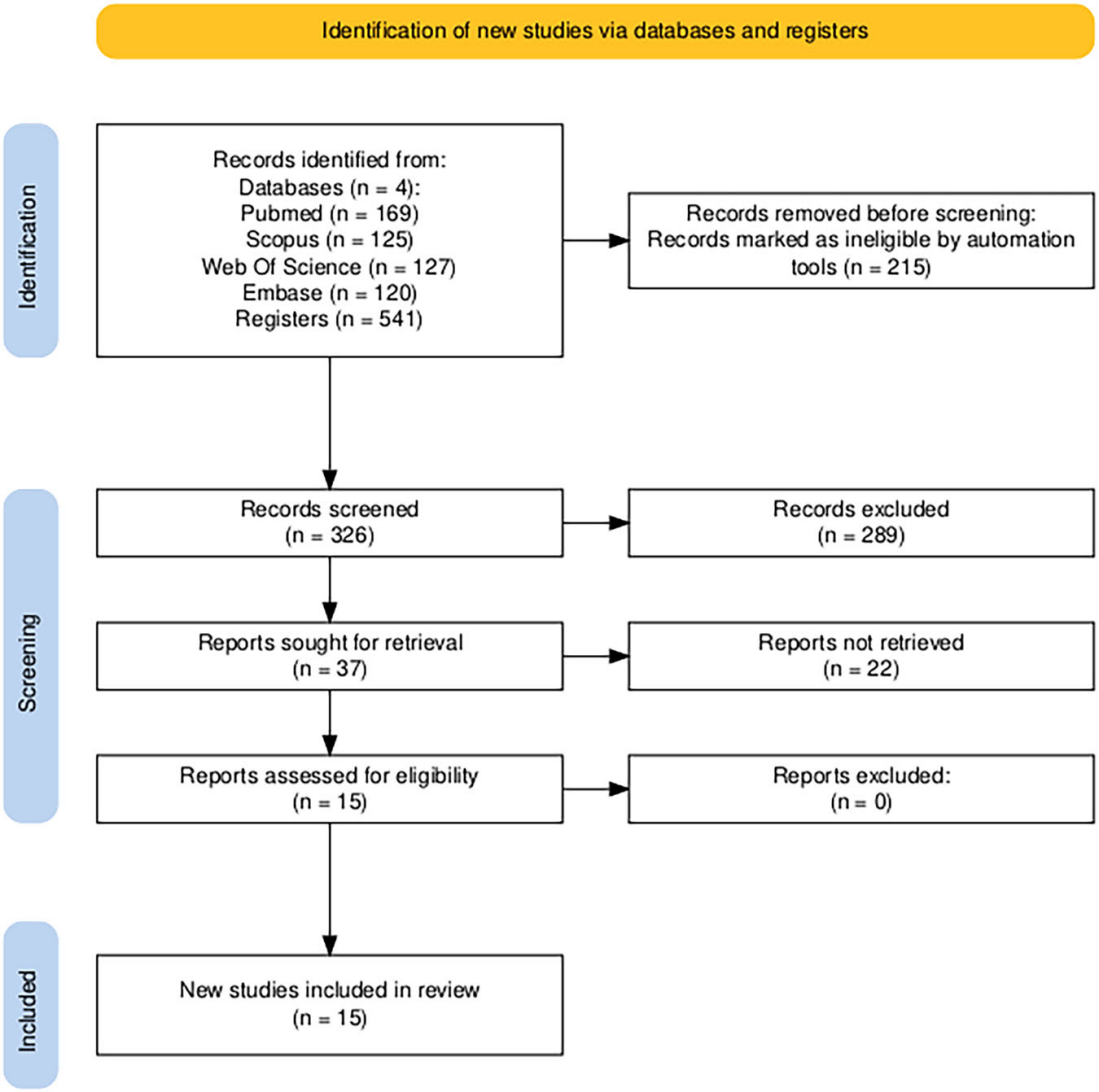
PRISMA 2020 flow diagram of evaluated studies.

Initially, 1027 articles were identified through database searches (PubMed, Scopus, Embase and Web of Science). After applying the eligibility criteria, which included language restrictions (English), and intervention type (exergame), a total of 563 were deemed relevant for further screening. Using Rayyan, references were managed, screened, and deduplicated. The inter-rater agreement for study selection was 79%, as estimated by Rayyan. Three hundred and nineteen duplicates out of 563 imported for screening were identified and removed. Following the title and abstract screening, 244 studies were selected for full-text analysis. However, 146 articles were excluded due to language (not in English), full text not available despite repeated attempts to retrieve it, irrelevant research focus (analyzing only motor or only cognitive outcomes), inappropriate population (e.g., children or healthy adults), or unsuitable study design (e.g., systematic reviews or meta-analyses). This systematic review ultimately included 11 studies, consisting of 8 randomized controlled trials (RCTs), 2 feasibility and usability studies, and 1 secondary analysis (Table 1). Study selection followed PRISMA item 17 recommendations and is illustrated in the flow diagram (Fig. 1 and Supplementary Table 3). The outcome measures and efficacy results of the included studies are summarized in Table 2.

**Table 1:** Characteristics of included studies

Type of disease	Author information	Type of study	Sample characteristic	Device	Training protocols	Treatment duration; location supervision
MCI	Saeed et al.^22^	RCT	*N* = 97; adults with mild cognitive impairment (MCI); age: 60–80 years	Custom exergame with wobble board (Android-based)	3 sessions/week, 40 min/session for 8 weeks; different difficulty levels (mild, moderate, high) controlled by goal size and ball speed	Duration: 8 weeks; Location: rehabilitation center (Pakistan Railway Hospital, Rawalpindi); Supervision: physical therapist trained in exergaming
	Li et al.^25^	RCT	*N* = 232; Older adults with mild cognitive impairment (MCI) or dementia; mean age: ~73 years	Nintendo Switch – WarioWare: Move It!	2 sessions/week, 60 min/session, 12 weeks; motion-sensing exercises (waving, jumping, arm swinging, rotational movements)	Duration: 12 weeks; Location: rural LTCFs (Shanxi Province, China); Supervision: trained staff provided tailored assistance
	mNCD Manser and de Bruin^23^	RCT	*N* = 37; older adults with mild neurocognitive disorder (mNCD); mean age: 72.8 ± 9.0 years	“Brain-IT” exergame platform (Dividat Senso Flex, Polar HRV sensors)	12-week individualized motor-cognitive exergame training with HRV-guided resonance breathing; ≥5×/week, 24 min/session	Duration: 12 weeks; Location: home-based; Supervision: 19–24 sessions remotely supervised
	Swinnen et al.^24^	RCT	45 participants (mean age 84.7–85.3 years, MMSE score: 17.2 ± 4.5); gender: 35 women (77.8%), 10 men (22.2%); diagnosis: Alzheimer’s disease, vascular dementia, mixed forms; inclusion criteria: age ≥65 years, diagnosed with MNCD, able to perform standing exercises	Dividat Senso exergame system: pressure-sensitive step training platform	8 weeks, 3 sessions/week, 15 min of exergaming (intervention) Control: 8 weeks, 3 sessions/week, 15 min of listening to music videos Training adjusted based on individual capabilities	Duration: 8 weeks; Location: University Psychiatric Centre KUL, De Wingerd long-term care facility (Belgium); Supervision: individual sessions supervised by a physical therapist to ensure safety and comfort
	Manser et al.^30^	Feasibility and usability study	*N* = 18; Mean age: 77.3 ± 9.8 years; 44.4% female; Older adults with mild neurocognitive disorder (mNCD)	“Senso Flex” (Dividat AG)	12-week individualized training with exergame-based motor-cognitive exercises and HRV-guided resonance breathing; Sessions: ≥5×/week, 21 min/session	Duration: 12 weeks; Location: home-based; Supervision: 19–24 sessions supervised by study investigators
	Werner et al.^31^	Secondary analysis	*N* = 56; Older adults with mild-to-moderate dementia; mean age: 82.7 ± 6.2 years	Physiomat® (balance training system)	10-week, task-specific motor-cognitive training; 2 sessions/week, focused on postural control and cognitive tasks (Trail Making Test)	Duration: 10 weeks; Location: rehabilitation center; Supervision: qualified trainer
	Salisbury et al.^28^	RCT	*N* = 39; older adults with subjective cognitive decline (SCD); mean age: 74.6 ± 7.4 years; 69% female	BrainFitRx (Moai Technologies, LLC)	3 sessions/week for 12 weeks; Dual-task exergaming combining moderate-intensity cycling and cognitive training	Duration: 12 weeks; Location: home-based (Telerehabilitation); Supervision: remote supervision via Zoom by study interventionists
Stroke	Kannan et al.^27^	RCT	*N* = 24; chronic stroke survivors (PwCS); hemiparetic, ambulatory; age N/A	Wii Fit (Nintendo)	6 weeks of high-intensity, tapered balance training; 20 sessions total; dual-task training with exergames (Wii Fit) combined with cognitive tasks	Duration: 6 weeks; Location: rehabilitation facility; Supervision: study investigators
	Huber et al.^29^	Feasibility study	*N* = 13 (10 completed); chronic stroke patients (≥18 years); median age: 68 years	Dividat Senso (pressure-sensitive platform)	2 sessions/week for 8 weeks; Motor-cognitive exergame-based rehabilitation with personalized progression using an adapted taxonomy	Duration: 8 weeks; Location: Physiotherapy center and senior home (Zurich, Switzerland); Supervision: one-to-one by trained movement scientists
	Maier et al.^26^	RCT	*N* = 30; age 45–75; community-dwelling chronic stroke patients (>6 months post-stroke); MoCA <26; mild–moderate motor deficits; no severe cognitive or perceptual impairments	VR-based system (Rehabilitation Gaming System - RGS) with motion capture, avatars, and adaptive algorithms	Daily 30 min (3 VR tasks × 10 min); 5 days/week for 6 weeks; tasks include Star Constellations, Quality Controller, Complex Spheroids	Location: hospital-based; Supervision: supervised setup only (therapists only assisted technically, no therapeutic feedback). Training executed independently by participants
PD	Yun et al.^32^	Prospective, single-center, single-arm feasibility study	12 participants (mean age 73.83 ± 6.09 years)	HTC Vive Pro (HMD, controllers, trackers, Steam VR base station)	10 sessions, 2–3 times a week, 30 min per session Exergames: go/no-go punch game, go/no-go stepping game, number punch game	Duration: 10 sessions, total of 30 min per session; Location: Seoul National University Hospital, Seoul, Republic of Korea; Supervision: Supervised by an occupational therapist during the VR sessions

*MCI* mild cognitive impairment, *SCD* subjective cognitive decline, *MS* multiple sclerosis, *PD* Parkinson’s disease, *RCT* randomized controlled trial.

**Table 2:** Outcome measures and efficacy of included studies

Type of disease	Author information	Outcome measures and efficacy	Main findings	Adverse events and missing schedule therapy
MCI	Saeed et al.^22^	Montreal Cognitive Assessment (MoCA), Trail Making Test, Stroop Test, ADAS Word List, Digit Span, Counting Backward Test	Significant improvement in global cognition (*p* = 0.01), inhibitory control (*p* = 0.83), working memory (*p* = 0.48), and attention in all exergame groups compared to control; higher cognitive challenge led to greater clinical improvement	No major adverse events; some participants reported dizziness; attrition rate: 10%
	Li et al.^25^	Sit-and-reach test, shoulder flexibility test, trunk rotation flexibility, range of motion, motor coordination (figure-of-8 walk test), hand dexterity test, cognitive tests (CASI, MMSE, MoCA)	The results show significant group × time interactions across multiple physical flexibility assessments, including the remaining distance between the hands and toes during the forward bend (*p* < 0.001), the distance between the hands clasped behind the back (*p* = 0.04), and the angle formed by trunk rotation to the left and right (*p* < 0.001). Significant group × time interactions also emerged for shoulder joint forward flexion (*p* < 0.001), abduction (*p* = 0.004), and elbow flexion (*p* = 0.049). In addition, the time to complete the figure-of-8 walk test (*p* < 0.001) and the number of blocks moved within 1 min (*p* = .02) showed significant interactions	No major adverse events; high adherence; no dropouts
	Manser and de Bruin^23^	Quick Mild Cognitive Impairment screen (QMCI), Learning & Memory tests (WMS-IV-LM), Executive Function (Trail Making Test, Go/No-Go), Gait Analysis, HRV metrics	Significant improvement in global cognition (large effect size) and in learning and memory (*p* = 0.022), immediate and delayed verbal recall; 55% of participants showed clinically relevant improvement	No major adverse events; attrition rate: 10%; technical issues led to some missing training sessions
	Manser et al.^30^	System Usability Scale (SUS), Exergame Enjoyment Questionnaire (EEQ), Behavioral Regulation in Exercise Questionnaire (BREQ); pre–post measurements for cognitive function, gait, and heart rate variability	Training was feasible and well-accepted; high adherence (85%), good usability (SUS = 71.7), and increased motivation (*p* = 0.03); the intervention group had an attrition rate of 20% and mean adherence and compliance rates of 85.0 and 84.1%, respectively. Preliminary data suggest cognitive and physical benefits	Three minor adverse events (falls with bruises, but no serious injuries); attrition rate 20%; some technical issues with the device
	Swinnen et al.^24^	Improved outcomes in exergame group: gait speed (SPPB), cognitive function (MoCA), step reaction time (SRTT), reduced depression (CSDD) No significant differences in neuropsychiatric symptoms (NPI), quality of life (DQoL), and ADL Attendance: exergame group 82.9%, control group 73.7%	Compared to the control group, the experimental group had significantly better gait speed after the intervention (*p* < 0.001); participants in the experimental group also had an overall better lower extremity functioning (*p* < 0.001) post-intervention and MoCA scores (*p* = <0.001). In addition, CSDD scores improved significantly after 8 weeks of exergame training (*p* < 0.001). There were no significant differences in NPI (*p* = 0.165), DQoL (*p* = 0.012), and ADL (*p* = 0.008) post-intervention scores between the experimental and control groups	No adverse events reported. Some participants dropped out due to transfers or premature study termination (COVID-19)
	Werner et al.^31^	Time required to complete Physiomat® tasks, Trail Making Test, Timed Up and Go, Dual-task cost (walking while counting)	Significant improvements in exergame performance within 3 weeks; participants with lower initial performance and visuospatial/divided attention deficits showed the fastest improvements. Post hoc analyses revealed significant improvements from T1 to TS7 in all Physiomat® tasks (*p* ≤ 0.001–0.006). Over the last two-thirds of the intervention period (TS7 to T2), the performance significantly improved across all Physiomat® tasks (*p* ≤ 0.001–0.036)	Four dropouts due to medical issues or lack of motivation; no major adverse events
	Salisbury et al.^28^	NIH Toolbox Cognitive Battery, 6-Min Walk Test (6MWT), Shuttle Walk Test (SWT)	Significant improvement in fluid cognition within the exergame group (exergame vs aerobic exercise only MD–SE = 0.7–108); no significant between-group differences in cognition or aerobic fitness	No major adverse events; 10.8% attrition rate due to COVID-19 shutdown; high adherence (85.6%)
Stroke	Kannan et al.^27^	Balance: Limits of Stability (LOS), Slip-Perturbation Test (SPT); Cognition: Letter-Number Sequencing (LNS)	For the LOS test under dual-task conditions, it was revealed no significant main effect of either time (*p* > 0.05) or group (*p* > 0.05), but there was a significant time x group interaction (*p* < 0.05) for cognitive accuracy on the letter number sequencing (LNS). The LNS cognitive task during the SPT resulted in significantly greater post-training accuracy for the experimental group than for the control group (*p* < 0.05)	No adverse events reported; no major dropouts; some missing data due to technical issues
	Huber et al.^29^	Feasibility (adherence, compliance, motivation, satisfaction), mobility (Timed-Up-and-Go, gait analysis), Cognition (TMT, MRT, TAP tests)	Feasible intervention with high adherence (95%) and motivation (77%); significant improvement in TUG (*p* = 0.05, *r* = 0.46); medium effect sizes for cognitive improvements (TMT, MRT)	One intervention-related adverse event (increased head tremor), one flu-related dropout; attrition rate: 23%
	Maier et al.^26^	Neuropsychological test battery (attention, memory, executive function, spatial awareness); HAM-D; MoCA; MMSE; Barthel Index	Significant improvements in attention (*p* < 0.01), spatial awareness (*p* < 0.01), and general cognition (*p* < 0.001) in the experimental group. For the control group, no significant change over time was found. Depression scores also improved in the experimental group only. Statistically significative *p* value has been registered only in the experimental group comparing value from T0 to T2 in attention (*p* = 0.01), spatial awareness (*p* = 0.00), and general cognitive functions (*p* = 0.00)	No adverse events reported. Minimal dropout (3/38), high adherence. One participant did fewer tasks
PD	Yun et al.^32^	Improvement in BBS and Stroop color–word test (*p* = 0.047, *p* = 0.003); no significant change in TUG, dual-task performance, or UPDRS	VR exergames led to improvement in executive function (Stroop test; *p* = 0.003) and balance (BBS; *p* = 0.047); participants demonstrated high satisfaction (6.0 on a 7-point Likert-type scale) and a low rate of adverse events (only mild blurred vision reported)	One adverse event: mild blurred vision (resolved the following day). No missing sessions reported

### Quality of the included studies—risk of bias

**Fig. 2:**
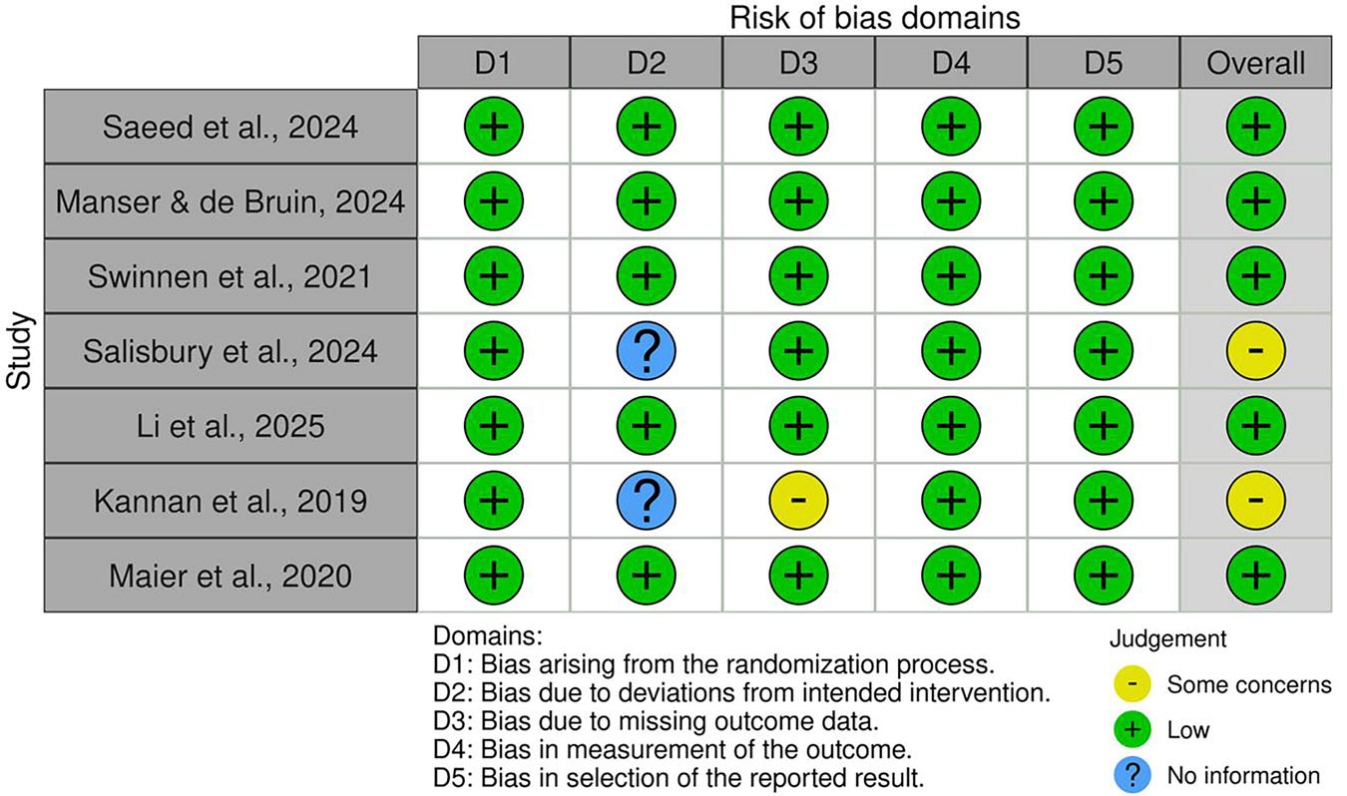
Cochrane risk of bias 2 (RoB 2). RoB 2 was used to evaluate bias across five domains: (1) bias arising from the randomization process, (2) deviations from intended interventions, (3) missing outcome data, (4) measurement of outcomes, and (5) selection of reported results. Each study will be classified as having low risk, some concerns, or high risk of bias.

**Fig. 3:**
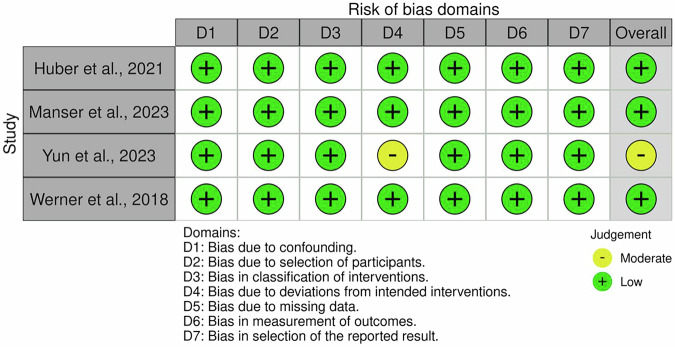
Risk of Bias in Non-randomized Studies of Interventions (ROBINS-I). ROBINS-I was applied to assess confounding, selection bias, classification of interventions, deviations from intended interventions, missing data, measurement of outcomes, and selective reporting. Studies will be rated as low, moderate, serious, or critical risk of bias.

Risk of bias was evaluated using the Cochrane RoB 2 tool for RCTs (Fig. 2) and the risk of bias in non-randomized studies of interventions (ROBINS-I) tool for non-randomized studies (Fig. 3). Among the eight RCTs, five were rated as low risk of bias across all domains, indicating high methodological quality^22–26^. However, two studies^27,28^ were judged as having “some concerns,” primarily due to insufficient information regarding deviations from intended interventions (domain 2), and in the case of Kannan et al.^27^, also due to missing outcome data (D3).

Regarding non-randomized studies, three out of four^29–31^ were rated as low risk. In contrast, the study by Yun et al.^32^ was classified as having moderate risk due to potential bias in outcome measurement and absence of blinding procedures. Full assessments are visually summarized in Figs. 2 and 3.

These findings suggest that the overall risk of bias across the studies included was acceptable, with a predominance of low-risk ratings. Nonetheless, the presence of reporting gaps and methodological limitations in a subset of studies was considered during the GRADE evaluation and contributed to downgrading the certainty of evidence for some outcome domains, particularly those related to feasibility and long-term effectiveness.

### Certainty of evidence

The certainty of evidence was evaluated using the GRADE approach and is summarized in Supplementary Table 2. For cognitive outcomes, the overall certainty was rated as moderate, based on a generally low risk of bias and minor inconsistency, although some studies presented imprecision due to small sample sizes and wide confidence intervals. For motor outcomes, the certainty was also rated as moderate, with no serious concerns regarding risk of bias or indirectness, but some inconsistency in the motor assessments used and variability in sample characteristics. Regarding feasibility and usability, the overall certainty was judged as low, primarily due to the qualitative nature of the data, smaller sample sizes, and variability in reporting across studies. Publication bias was considered unlikely in all domains.

### Data synthesis

As per PRISMA item 20 and SWiM reporting guidelines, data were synthesized narratively given the heterogeneity of interventions, populations, and outcomes. Across the included studies, substantial heterogeneity emerged in intervention parameters (e.g., session frequency, intensity, and duration), participant characteristics (e.g., diagnosis, age, baseline cognitive status), and outcome measures. Cognitive assessments ranged from global cognition tools (Montreal Cognitive Assessment (MoCA), Mini-Mental State Examination (MMSE) and Cognitive Abilities Screening Instrument (CASI) to domain-specific tasks (e.g., Stroop, Trail Making Test (TMT), Digit Span), while motor outcomes varied from functional mobility tests (e.g., Timed-Up-and-Go (TUG), BBS) to coordination and aerobic measures. This diversity, while reflecting the ecological applicability of exergames, limits direct comparisons and emphasize (refer to Supplementary Table 4).

### Mild cognitive impairment (MCI), mild neurocognitive disorder (mNCD), and mild-to-moderate dementia

Seven studies investigated the effects of exergame-based interventions in populations with MCI, mNCD, or mild-to-moderate dementia. Of these, five were RCTs^22–25,28^, one was a feasibility study^30^, and one was a secondary analysis^31^. The duration of the intervention ranged from 8 to 12 weeks, with session frequencies ranging from 2 to 5 times per week. Most interventions used commercial or custom-developed platforms (e.g., Nintendo Switch, Dividat Senso, BrainFitRx), often targeting motor and cognitive components through dual-task paradigms. Settings included long-term care facilities^24,25^, rehabilitation centers^22,31^, and remotely supervised home-based programs^28,30^. Sample sizes ranged from 18^30^ to 232^25^.

Based on cognitive outcomes, most studies reported positive effects of exergames on global cognition and executive functions. Saeed et al.^22^ observed significant improvements in several domains (MoCA, Stroop, TMT, Digit Span), with higher difficulty levels leading to greater cognitive gains. Li et al.^25^ found improvements in general cognitive performance as measured by CASI, MMSE, and MoCA. Manser and De Bruin^23^ reported large effect sizes in global cognition and verbal recall (WMS-IV-LM), with 55% of participants showing clinically significant changes. Swinnen et al.^24^ demonstrated improvements in MoCA scores in dementia patients. Werner et al.^31^ found that task performance and TMT scores improved significantly within three weeks, especially in individuals with underlying attention deficits. In contrast, Salisbury et al.^28^ reported within-group improvements in fluid cognition, but no significant differences from control. Overall, the results suggest that exergaming may improve multiple cognitive domains in older adults with MCI or dementia, although effect sizes and domains targeted varied across studies.

Regarding motor outcomes, motor improvements were consistently reported across studies. Li et al.^25^ showed increases in flexibility, coordination, and manual dexterity. Swinnen et al.^24^ observed improvements in gait speed and step reaction time, and Werner et al.^31^ showed improved balance and performance in two tasks (pulling and walking with counting). Although less detailed, Saeed et al.^22^ and Manser and De Bruin^23^ also included gait or balance parameters. Salisbury et al.^28^, using the 6-minute walking test and shuttle walking test, found no significant changes in aerobic fitness, likely due to ceiling effects in community-dwelling older adults. Despite methodological variability, these studies support the motor efficacy of exergames in cognitively vulnerable older populations.

In terms of feasibility and usability, all included studies reported high adherence, ranging from 82.9% to 100%. No serious adverse events were reported, except for mild dizziness^22^ and some non-injurious falls^30^. Usability scores were high (SUS > 70 in Manser et al.^30^), and participants frequently reported enjoyment and satisfaction. Remote supervision was successfully implemented in Manser et al.^30^ and Salisbury et al.^28^, supporting the feasibility of home-based telerehabilitation programs. Some discontinuation was observed in Swinnen et al.^24^ and Salisbury et al.^28^ due to COVID-related issues, but no dropouts were attributed to the difficulty or usability of the intervention.

### Stroke

Three studies have explored the impact of exergame-based interventions in individuals with chronic stroke^26,27,29^. Two were RCTs^26,27^ and one was a feasibility study^29^. Interventions ranged in duration from 6 to 8 weeks, with training frequencies from 2 to 5 sessions per week. Devices used included commercial systems such as Nintendo Wii Fit^27^, pressure-sensitive platforms with personalized progression^29^, and VR rehabilitation systems with adaptive algorithms^26^. Settings ranged from rehabilitation facilities and hospitals to senior centers, with sessions typically supervised or semi-supervised.

Cognitive outcomes were assessed in all three studies, although using heterogeneous measures. Maier et al.^26^ reported significant improvements in attention, spatial awareness, and general cognitive ability (MoCA and MMSE) in the experimental group, but no significant changes in executive function. Kannan et al.^27^ found improved cognitive performance in dual-task conditions, assessed by the Letter-Number Sequencing Test. Huber et al.^29^ reported medium effect sizes for cognitive gains in attention and processing speed, assessed by the TMT, the Mental Rotation Task, and the Test of Attentional Performance. Overall, these findings support the potential of exergames to improve attention and cognitive flexibility in patients with chronic stroke, although evidence on executive function remains mixed.

Motor outcomes were consistently positive. Kannan et al.^33^ demonstrated significant improvements in volitional and reactive balance in stability and glide-perturbation tests, favoring exergame training over conventional rehabilitation. Huber et al.^29^ observed significant improvement in the TUG test (*p* = 0.05, *r* = 0.46) and favorable changes in gait analysis. Maier et al.^26^ reported improvements in general mobility (Barthel index) with cognitive changes. These findings suggest that exergames, particularly those with a motor-cognitive component, may significantly contribute to balance, mobility, and functional independence in stroke rehabilitation.

Feasibility and usability were also encouraging. Adherence rates were high across all studies (95% in Huber et al.^23^; minimal dropout in Maier et al.^26^), and participants reported high motivation and satisfaction. No serious adverse events were recorded. Mild issues included technical difficulties^27^ and an increase in intervention-related cranial tremor^29^. Overall, the interventions were well tolerated and adaptable to different rehabilitation settings.

### Parkinson’s disease

One study investigated the effects of exergaming in individuals with Parkinson’s disease. Yun et al.^32^ conducted a single-arm feasibility study involving 12 participants who engaged in a VR-based exergaming program using the HTC Vive Pro. The intervention consisted of 10 sessions, delivered 2–3 times per week, each lasting 30 min, and included go/no-go punching and stepping games as well as number-based motor-cognitive tasks. All sessions were supervised by an occupational therapist at a hospital-based setting.

Regarding cognitive outcomes, participants showed significant improvement in executive function as assessed by the Stroop color–word test (p = 0.003). In terms of motor outcomes, significant improvements were observed in balance, measured by the Berg Balance Scale (*p* = 0.047), whereas no significant changes were found in TUG performance, dual-task outcomes, or UPDRS scores. The intervention was well tolerated, with high participant satisfaction and no serious adverse events reported. Only one case of mild blurred vision was recorded, which resolved spontaneously the next day, and no missed sessions were reported.

## Discussion

As far as we know, this is the first review deeply investigating the potential role of exergaming in improving motor-cognitive functions in neurological patients. This systematic review included 11 studies: 8 RCTs, 2 feasibility and usability studies, and 1 secondary analysis, investigating the effects of exergame-based interventions on combined cognitive and motor outcomes in neurological populations. Despite the heterogeneity of populations and protocols, most studies reported improvements in executive function, global cognition, attention, and memory, along with motor improvements in balance, gait, and functional mobility. These improvements were generally accompanied by high adherence and positive usability ratings, suggesting that exergames are effective and well tolerated. Then, evidence suggests their promise as a structured, engaging, and scalable neurorehabilitation tool.

Across all studies reviewed, exergames demonstrated consistent positive effects on cognitive domains, particularly executive function, global cognition, and memory, as per the standardized tool used, such as MoCA, CASI, Stroop, and TMT^22,23,25^. These results are consistent with previous evidence highlighting the neuroplastic potential of dual-task paradigms, which stimulate the prefrontal cortex and other integrative neural circuits involved in cognition. However, the extent of improvement varied depending on participant characteristics, intervention intensity, and baseline cognitive status. For example, Salisbury et al.^28^ observed within-group improvements with no significant effects compared to the control group, highlighting the possible moderating role of initial cognitive reserve. Moreover, Maier et al.^26^ found improvements in attention and spatial awareness, but not in executive functions, highlighting the importance of task-specific design for the results. These findings further support the importance of tailoring interventions to cognitive profiles while underscoring the need for standardized cognitive outcome sets across studies.

Concerning motor function, most studies reported improvements in balance, mobility, flexibility, and motor coordination. Interventions targeting dual-task performance^24,27^ were particularly effective in improving gait and postural control. In fact, neurological patients, especially those affected by stroke and MCI, presented significative improvements in Timed Up and Go (TUG), Figure 8, and Step Reaction Time^25,31^. On the other hand, Salisbury et al.^28^ did not find significant changes in aerobic capacity, likely due to ceiling effects in physically active older adults. These results suggest that motor outcomes are influenced by both intervention parameters and individual physical status, underscoring the need to adapt train intensity and task progression.

Exergames showed high levels of adherence, with completion rates ranging from 82.9% to 100%. No serious adverse events were reported, with only isolated cases of dizziness or mild discomfort^29^. Usability was generally rated positively (e.g., SUS > 70), and qualitative reports often emphasized user enjoyment and satisfaction. Importantly, remote supervision^28,30^ was found feasible and well accepted, supporting the integration of telerehabilitation models. However, some studies lacked systematic feasibility metrics, and adherence definitions varied. Our GRADE assessment, informed by RoB 2 and ROBINS-I evaluations, confirmed an overall low to moderate risk of bias across the included studies. While the majority of randomized and non-randomized trials showed low risk in key domains, some studies presented limitations related to adherence tracking, incomplete outcome data, and lack of protocol registration. These factors, combined with moderate heterogeneity in intervention design, outcome measures, and sample sizes, contributed to a downgrading of the certainty of evidence, particularly for feasibility and usability outcomes. Despite the generally high adherence and positive usability reported, the certainty of evidence for these domains was rated as low, largely due to the absence of standardized reporting practices and the reliance on small samples or qualitative evaluations. Importantly, the observed heterogeneity reflects the early stage of implementation of exergaming in clinical practice, and it underlines the need for harmonized study protocols tailored to specific neurological profiles. To enhance the robustness and comparability of future findings, upcoming trials should adopt rigorous designs, larger samples, and validated tools for assessing feasibility, usability, and safety. Pre-registration and transparent reporting will also be essential to reduce the risk of publication and reporting biases.

Overall, the current evidence suggests that exergames represent a promising, engaging, and adaptable tool for cognitive-motor neurorehabilitation in diverse neurological populations. While improvements in executive function, attention, balance, and gait were consistently reported, the variability in effect sizes highlights the importance of intervention customization and the need for further confirmatory trials with high methodological rigor.

A major issue in exergaming research is the inconsistent classification of digital interventions. The frequent mislabelling of exergames, serious games, and VR-based rehabilitation tools has introduced confusion in systematic reviews and meta-analyses, ultimately compromising the accuracy of efficacy estimates. This ambiguity also impacts the development of regulatory frameworks and hinders the clinical adoption of these technologies.

**Fig. 4:**
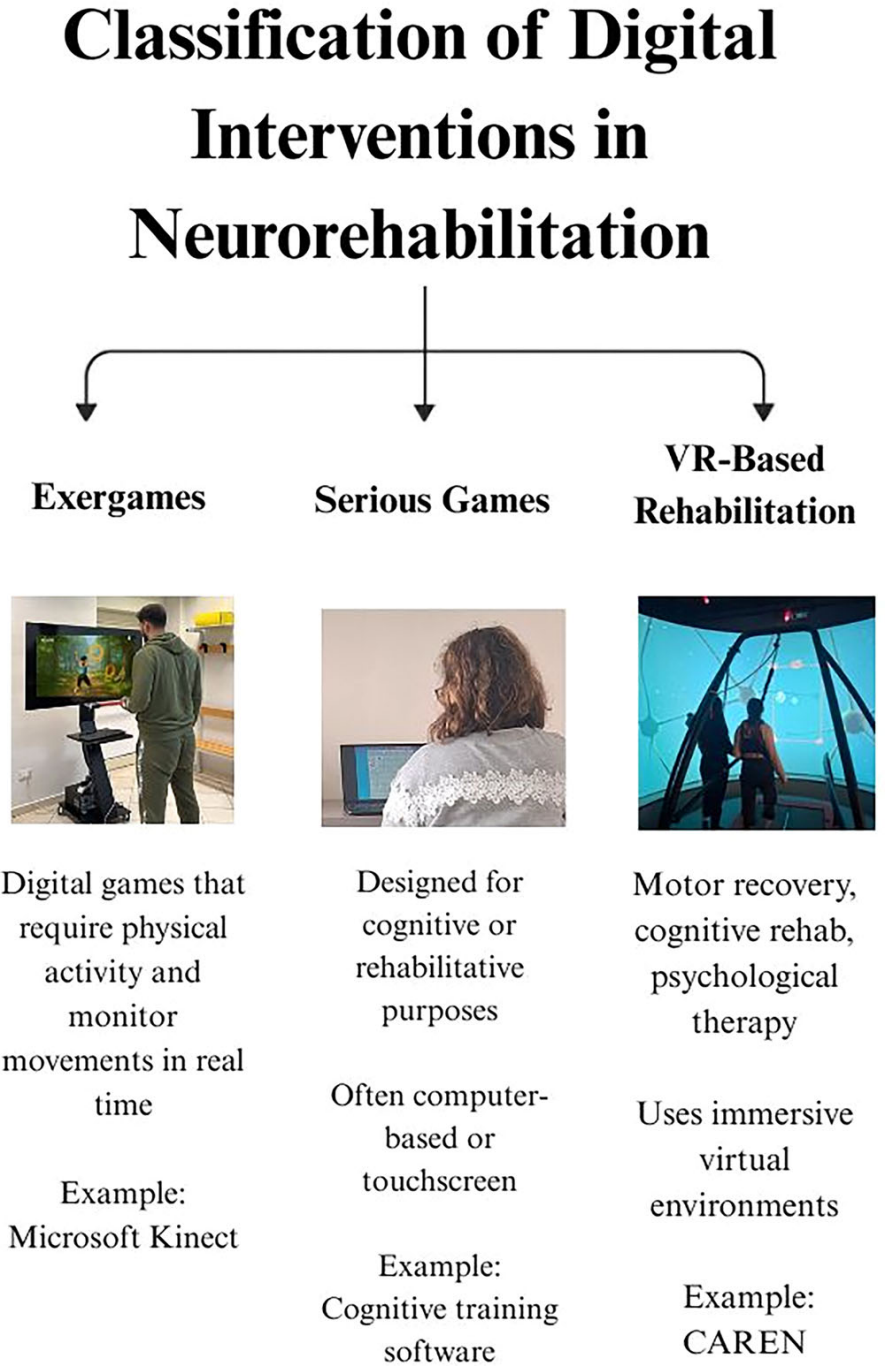
Typology of digital tools for neurorehabilitation: a visual framework.

To provide a clearer understanding of the distinctions between these digital interventions, Fig. 4 and Table 3 summarize their key characteristics, therapeutic applications, and primary targets. This classification helps differentiate exergames from serious games and VR-based rehabilitation, avoiding conceptual misinterpretations that could impact research synthesis and clinical implementation.

**Table 3:** Classification of digital interventions in neurorehabilitation

Category	Definition	Main characteristics	Primary target	Examples
Exergames	Digital games that require active full-body movement, integrating motor and cognitive tasks	Motion-tracking sensors (e.g., Kinect, Wii, VR controllers) Engages motor and cognitive functions simultaneously Real-time feedback to improve performance	Motor rehabilitation (balance, gait, coordination), dual-task training	Wii Fit, Kinect Sports
Serious games	Games designed primarily for cognitive engagement and behavioral training rather than physical movement	Limited or no movement required Often used for cognitive rehabilitation, memory training, and problem-solving tasks May involve touchscreen or computer-based tasks	Cognitive rehabilitation, behavioral modification, psychological therapy	BrainHQ, Cogmed
VR-based rehabilitation	Rehabilitation that uses virtual environments to provide immersive therapy, with active or passive engagement	Active VR: patient interacts with the environment (e.g., reaching, stepping, navigating a VR space). Passive VR: the environment changes without requiring movement (e.g., virtual scenery for relaxation or cognitive therapy)	Motor recovery, cognitive training, sensory-motor integration	CAREN system, VR-based mirror therapy

Exergames, by definition, require physical movement as a central component of gameplay, using motion-sensing interfaces to provide real-time feedback and progressive task adaptation^34^. In contrast, serious games prioritize cognitive engagement rather than physical activity, typically focusing on skill-based learning or behavior modification^35^. While some serious games involve light physical interaction, movement is not the primary therapeutic mechanism, which distinguishes them from exergames.

A further distinction within exergames themselves lies in their underlying design principles. Some exergames are structured to closely resemble gym-based rehabilitation, focusing on repetitive, standardized exercises that target specific motor functions. These interventions typically provide clear performance metrics, making them more suitable for clinical integration. In contrast, other exergames are more game-oriented, prioritizing engagement through interactive challenges, narrative-driven gameplay, or competitive elements. While these can enhance motivation and adherence, they may offer less precise clinical feedback. From a rehabilitation standpoint, the choice between these two types of exergames should be guided by the specific therapeutic goals, the patient’s cognitive and motor profile, and the need for accurate outcome measurement. As previously noted by Maldonado Diaz et al.^36^, the lack of uniformity in software design and the variability in data reporting between these different types of exergames pose challenges for clinicians seeking to integrate them into structured rehabilitation programs. Establishing clearer taxonomies and validating standardized performance metrics would help bridge this gap, ensuring that both clinically focused and entertainment-based exergames can be effectively utilized in neurorehabilitation.

VR introduces additional classification challenges. Unlike exergames or serious games, VR is not an intervention type but rather a technological medium that can be applied across both domains. VR-based rehabilitation can range from fully immersive (headset-based) to semi-immersive (projection-based) or non-immersive (screen-based), with varying levels of motor engagement^37^. VR-exergames integrate full-body movement within an immersive setting, whereas VR-serious games focus on cognitive challenges such as memory, problem-solving, or exposure therapy^30^. A critical issue is that many meta-analyses have grouped exergames with serious games or VR-based interventions, potentially inflating or underestimating therapeutic effects^38,39^. This raises concerns about the validity of previous systematic reviews and meta-analyses, as they may have drawn conclusions based on heterogeneous intervention types. Moving forward, clear taxonomies should be established to ensure interventions are correctly classified and compared. Furthermore, this conceptual inconsistency has implications for funding policies, insurance reimbursement, and clinical guideline development. If regulatory agencies fail to differentiate active motor-based interventions from passive cognitive ones, reimbursement pathways may unintentionally exclude effective exergaming therapies. Policymakers and research funding bodies should develop intervention-specific guidelines to standardize classification and ensure accurate reporting in clinical trials. Only through intervention-specific guidelines and rigorous classification can future trials ensure accurate reporting, valid comparisons, and optimized implementation.

Despite compelling evidence supporting their efficacy, exergames remain underutilized in clinical rehabilitation due to multiple barriers. A primary limitation is the absence of standardized dosing guidelines for different neurological conditions, particularly regarding session frequency, intensity, and duration. While some studies report that 4–5 sessions per week enhance cognitive-motor outcomes, others suggest that excessive training loads may decrease adherence^40^. This inconsistency complicates clinical decision-making and hinders widespread adoption.

Another key issue is the lack of harmonized outcome measures. Although tools like MoCA and TMT were frequently used to assess cognition, as well as TUG and BBS for motor function, considerable variability remains. While tailoring assessments to patient populations is crucial (e.g., QMCI for MCI), such variability reduces comparability across studies. Future research should define core outcome sets validated per condition, enabling both patient-centered assessments and cross-study comparisons. This balance between flexibility and standardization is essential. Implementation challenges also include software variability, tracking accuracy, and clinician expertise, which affect adherence and intervention fidelity, particularly in individuals with visuospatial or cognitive deficits^36^.

Beyond outcome measures, inconsistencies in treatment frequency, duration, intensity and supervision further hinder the development of standardized guidelines. The reviewed studies implemented intervention periods ranging from two to twelve weeks, with some adopting high-frequency regimens (5x/week) and others opting for lower frequencies (2–3×/week). Manser et al.^30^ and Sturnieks et al.^41^ suggest that structured, high-frequency protocols with adaptive feedback yield better outcomes. However, intensity was often not reported or standardized, limiting replicability. Huber et al.^42^ highlighted the value of real-time personalization based on cognitive-motor profiles, reinforcing the need for individualized but structured approaches.

Intensity, a critical variable to maximize exercise effectiveness in cognitive, motor, and neuroplasticity outcomes, was not standardized and/or described between the included studies^43^. This limits the ability to replicate findings and to define optimal intervention thresholds. Huber et al.^42^, for instance, proposed a personalized model for stroke patients, adjusting difficulty and feedback in real time based on cognitive-motor profiles. These findings reinforce that individualized, high-frequency protocols are more likely to produce meaningful improvements.

Furthermore, exergames are rarely integrated into multimodal rehabilitation models, despite evidence suggesting that combining them with conventional physiotherapy or cognitive therapy enhances therapeutic outcomes^27^. Future studies should explore hybrid rehabilitation models, evaluating whether exergames are most effective as first-line therapy, adjunct therapy, or post-discharge maintenance therapy.

Patient variability also influences efficacy. Neurodegenerative patients may benefit from simplified interfaces, while stroke survivors may need targeted motor relearning tasks. Previous physical activity, visuospatial deficits, or cognitive rigidity can affect engagement, necessitating adaptive design. Studies support the importance of usability, intuitive interfaces, and personalized feedback in enhancing motivation and usability^23,38,44^. User experience is also crucial. Factors like ease of use, cognitive load, and interface complexity impact engagement, especially in older adults. Issues such as motion sickness or avatar confusion can reduce adherence^45–49^. Future studies should assess usability systematically, beyond adherence alone^45–47^.

Finally, tele-exergaming offers scalability, particularly for patients with limited access. However, barriers such as technology access, internet stability, and safety monitoring remain. Broader adoption will require digital health policies addressing training, reimbursement, and integration into care frameworks^28^.

In summary, current evidence supports the use of exergames as a complementary neurorehabilitation strategy, optimally delivered 2–3 times per week over 6–8 weeks, especially when applied in a structured, population-specific manner^23,41^. Future trials should compare exergames with conventional therapies, include long-term follow-up, and integrate neurophysiological markers to inform policy and clinical practice. Future RCTs should directly compare exergames with conventional and digital therapies, including long-term follow-up and neurophysiological markers, to inform implementation and policy guidelines.

To establish exergames as a gold-standard rehabilitation tool, several critical advancements are required. Health policies should increasingly support the integration of exergames as distinct, reimbursable interventions, especially when tailored to specific neurological conditions.

Another major research priority is the development of neurophysiologically validated protocols. While current evidence suggests that exergames improve cognitive and motor functions, a critical gap in the literature is the lack of direct head-to-head comparisons between exergames, serious games, and traditional cognitive or motor training tasks. Without RCTs directly comparing these approaches, it remains unclear whether exergames provide superior benefits over conventional training methods or whether their advantages stem primarily from increased engagement and motivation^23,41^. Future studies should aim to isolate these factors by designing protocols that systematically compare exergame-based interventions with both conventional therapies and other digital approaches. Such trials would clarify the mechanisms driving observed benefits and inform clinical decision-making^50^.

Future implementation of exergames should focus on their integration within multimodal rehabilitation frameworks, emphasizing adaptable, patient-centered protocols tailored to cognitive and physical abilities to maximize therapeutic outcomes. Furthermore, widespread adoption will require investment in clinician training, ensuring that healthcare professionals possess the necessary expertise to implement exergame-based interventions effectively.

Interdisciplinary collaboration will be essential to refine intervention design, validate neurophysiological mechanisms, and support regulatory recognition. O’Neil et al.^48^ and Perez-Narcos et al.^49^ emphasize that the successful adoption of VR and exergames in neurorehabilitation requires alignment between technological innovation and clinical applicability. Furthermore, insights from clinical implementation studies highlight the necessity of standardized protocols by neurological profile, usability assessments, and the development of reimbursement policies that support exergame-based rehabilitation^38^.

With continued research, policy support, and structured clinical frameworks, exergames could evolve into a standard, scalable, and neurophysiologically grounded solution for cognitive-motor rehabilitation.

This systematic review provides a comprehensive synthesis of the existing literature on exergames as a cognitive-motor rehabilitation tool, highlighting their potential to improve executive function, working memory, balance, gait, and mobility across neurological and aging populations. By integrating findings from multiple RCTs, feasibility, quasi-experimental, and secondary-analyses studies, this review reinforces the growing scientific and clinical interest in exergames and their role in neuroplasticity-driven recovery. The inclusion of studies across multiple neurological conditions (MCI, dementia, stroke, multiple sclerosis, frailty, and balance impairments) strengthens the generalizability of the findings, emphasizing exergames’ versatility as an intervention across diverse patient populations. Furthermore, this review goes beyond summarizing findings, critically engaging with conceptual inconsistencies in the field by clearly differentiating exergames from serious games and VR-based rehabilitation, addressing a major gap in prior research syntheses.

Despite its strengths, this review has several limitations that should be acknowledged. First, heterogeneity in study design, intervention protocols, and outcome measures limits direct comparability between studies, making it difficult to establish a consensus on optimal intervention parameters (e.g., frequency, intensity, session duration) and limiting the possibility of conducting a meta-analysis. This variability also complicates the synthesis of effect sizes, particularly due to study heterogeneity. It undermines the statistical power of evidence comparison, reinforcing the importance of developing standardized outcome measures and harmonized intervention protocols for future research. Moreover, the variability in cognitive and motor assessments, including the use of different cognitive screening tools (MoCA, TMT, Stroop Test, ADAS), presents an additional challenge for cross-study comparisons. Another limitation is the lack of long-term follow-up data in many included studies, restricting conclusions about the sustained benefits of exergames beyond the immediate intervention period. The potential for publication bias also exists, as studies reporting positive results are more likely to be published, leading to possible overestimation of exergames’ effectiveness. Additionally, limited neurophysiological validation (e.g., functional magnetic resonance imaging (fMR)I, electroencephalogram (EEG)) in the reviewed studies hinders a deeper understanding of the underlying neural mechanisms driving cognitive-motor improvements.

Moreover, it is important to acknowledge that the majority of studies included in this review did not directly assess neurophysiological mechanisms through EEG, fMRI, or biomarker analyses. Therefore, the proposed mechanistic interpretations, such as brain-derived neurotrophic factor activation and cognitive-motor network coupling, remain theoretical and are based on evidence from adjacent research fields. Existing models suggest that exergaming may support neuroplasticity by enhancing brain connectivity and promoting synaptic growth, particularly in motor and executive function networks^27,37,51–53^. Nonetheless, these hypotheses require empirical validation through future studies that integrate objective neurophysiological assessments into trial design.

While randomized trials remain the gold standard for evaluating efficacy, incorporating findings from descriptive research can help address feasibility concerns and optimize implementation strategies. These studies provide valuable insights into real-world challenges such as patient adherence, software variability, and clinical integration, which are not always captured in controlled trials. Future research should aim to bridge the gap between efficacy-focused trials and feasibility studies to ensure that exergame-based interventions are adaptable to diverse clinical settings and patient needs.

Another limitation is the potential presence of publication bias. Although not formally assessed with statistical tools such as funnel plots, a qualitative examination suggested an overrepresentation of studies reporting positive findings and a lack of pre-registered protocols. This bias may have led to an overestimation of the efficacy of exergame interventions and was considered in the overall GRADE evaluation.

Finally, this review primarily focused on quantitative outcome measures, with fewer studies addressing patient-reported experiences, barriers to adoption, and qualitative insights into adherence and engagement. Future systematic reviews should consider including mixed methods analyses to better understand the psychological, behavioral, and motivational factors influencing exergame efficacy. Despite these limitations, this review provides a solid foundation for advancing research and clinical implementation in this rapidly evolving field.

This review shows the potential of exergames as a cognitive-motor rehabilitation tool, thanks to the enhanced engagement, superior adherence, and dual-task benefits over conventional therapies. By leveraging neuroplasticity through cognitive-motor coupling, task adaptability, and gamification, exergames provide a scalable and accessible intervention for neurological and aging populations. Nevertheless, for exergames to evolve into a standardized clinical tool, several critical steps are necessary, including the development of harmonized training protocols, long-term RCTs, and neurophysiological validation to consolidate their therapeutic value. A clear conceptual differentiation between exergames, serious games, and VR-based therapies is also essential to prevent misclassification in clinical guidelines and research synthesis. Notably, tele-exergaming should be further explored and integrated into digital healthcare frameworks, as it offers promising solutions for extending rehabilitation access to patients in remote or underserved areas. This requires addressing key issues such as technological infrastructure, clinician training, remote monitoring, and reimbursement models. If these implementation challenges are met, exergames, including their remote applications, can move beyond the experimental stage to become a standardized, evidence-based tool for enhancing functional recovery in neurological rehabilitation.

## Methods

This systematic review investigated the efficacy of exergame interventions in neurological and neurodegenerative populations. The review protocol was registered on PROSPERO (Registration ID: CRD420250655053), and it followed the Preferred Reporting Items for Systematic Reviews and Meta-Analyses (PRISMA) guidelines^54^ and the Cochrane Handbook for Systematic Reviews of Interventions^55^, and the Synthesis Without Meta-analysis (SWiM)^56^ reporting framework for narrative syntheses.

### Eligibility criteria and PICOS framework

This systematic review followed the PICOS framework to define eligibility.

Studies were included if they met the following criteria:

Population (P): Adults (aged ≥18 years) diagnosed with neurological or neurodegenerative conditions (e.g., stroke, Parkinson’s disease, MCI, dementia, multiple sclerosis).

Intervention (I): Exergame-based interventions, defined as digital applications combining physical exercise with gamified tasks. Any exergame platform was accepted, provided the intervention targeted rehabilitation goals.

Comparator (C): Valid comparators included standard physiotherapy, cognitive training, passive VR, other digital therapies, or no intervention. Studies using a pre-post design without an explicit comparator were also eligible.

Outcomes (O): To be included, studies had to report both cognitive and motor outcomes, even if the intervention was not explicitly dual-task or integrated. Cognitive outcomes included executive functions, global cognition, attention, or memory. Motor outcomes included balance, gait, mobility, or motor coordination. Secondary outcomes included functional, emotional, and adherence-related factors, such as quality of life, motivation, social engagement, and adherence rates (e.g., session completion or dropout).

Study design (S): Eligible designs included RCTs, non-RCTs, quasi-experimental designs, and feasibility or pilot studies. Descriptive cohort or cross-sectional studies without an intervention were excluded.

Studies focusing on pediatric populations, animal models, case reports, reviews, conference abstracts, protocols, or proof-of-concept pilots were excluded. Only full-text articles in English were considered.

### Search strategy

A comprehensive literature search was conducted in PubMed, Scopus, Embase, and Web of Science, including all studies published before April 2025. No trial registers were consulted, as only completed interventional studies were eligible for inclusion. To ensure a systematic and reproducible approach, a combination of Medical Subject Headings (MeSH) terms and free-text keywords was used. The following key MeSH terms and keywords were used:

1. (“motor function” OR “motor learning” OR “motor rehabilitation”) AND (“cognitive function” OR “executive function” OR “cognition” OR “dual task”) AND (“exergame”);
2. (“exergamers”[All Fields] OR “exergaming”[MeSH Terms] OR “exergaming”[All Fields] OR “exergame”[All Fields] OR “exergames”[All Fields]) AND ((“motor”[All Fields] OR “motor s”[All Fields]) AND (“functional”[All Fields] OR “functioning”[All Fields] OR “functions”[All Fields])) AND (“cognition”[MeSH Terms] OR “cognition”[All Fields] OR (“cognitive”[All Fields] AND “function”[All Fields]) OR “cognitive function”[All Fields]).
3. “Dual”[All Fields] AND “Task”[All Fields] AND (“exergamers”[All Fields] OR “exergaming”[MeSH Terms] OR “exergaming”[All Fields] OR “exergame”[All Fields] OR “exergames”[All Fields]);
4. (“motor function” OR “motor control” OR “motor learning” OR “motor rehabilitation”) AND (“cognitive function” OR “cognition” OR “dual task”) AND (“exergame”) AND (“neuroplasticity” OR “brain connectivity”);
5. (“Virtual Reality”[MeSH Terms] OR “Virtual Reality”[Title/Abstract]) AND (“Video Games”[MeSH Terms] OR “Serious Games”[MeSH Terms] OR “Games”[Title/Abstract]) AND (“Rehabilitation”[MeSH Terms] OR “Cognitive Therapy”[MeSH Terms] OR “Physical Therapy Modalities”[MeSH Terms] OR rehabilitation[Title/Abstract]) AND (“Motor Skills”[MeSH Terms] OR “Cognition”[MeSH Terms] OR “Dual Task”[Title/Abstract])
6. (“Telerehabilitation”[MeSH Terms] OR “Remote Consultation”[MeSH Terms] OR telerehabilitation[Title/Abstract]) AND (“Virtual Reality”[MeSH Terms] OR “Virtual Reality Exposure Therapy”[MeSH Terms] OR “Virtual Reality”[Title/Abstract]) AND (“Video Games”[MeSH Terms] OR “Exergames”[MeSH Terms] OR “Computer Simulation”[MeSH Terms] OR games[Title/Abstract]) AND (“Motor Activity”[MeSH Terms] OR “Motor Skills”[MeSH Terms] OR “Movement”[MeSH Terms] OR “motor function”[Title/Abstract]) AND (“Cognition”[MeSH Terms] OR “Cognitive Dysfunction”[MeSH Terms] OR “Cognition Disorders”[MeSH Terms] OR “cognitive function”[Title/Abstract]) AND (“Rehabilitation”[MeSH Terms] OR rehabilitation[Title/Abstract])

The complete and full search strings for each database are reported in Supplementary Table 1, in accordance with PRISMA guidelines (Item 8), to ensure transparency and reproducibility.

### Study selection and data extraction

The study selection process adhered to the PRISMA 2020 guidelines and was conducted using a blinded approach via Rayyan, a web-based tool for systematic reviews. Before importing references into Rayyan, a preliminary screening was performed to exclude studies that clearly did not meet the basic eligibility criteria based on the PICOS framework (e.g., non-English language, animal studies, and non-interventional publication types such as reviews, protocols, and conference abstracts). In Rayyan, two independent reviewers (R.M., G.P.) screened titles and abstracts in a blinded manner. In the second phase, full-text articles were assessed using the same eligibility criteria to determine final inclusion. During the full-text screening phase, all efforts were made to retrieve unavailable articles, including searching institutional databases and contacting corresponding authors when necessary. Articles for which the full text could not be retrieved despite these attempts were excluded from the final analysis. After unblinding, any disagreements were resolved through discussion. When consensus could not be reached, a third reviewer (M.G.M.) was involved to adjudicate and finalize inclusion decisions. The full study selection process is illustrated in Fig. 1 (PRISMA 2020 flow diagram). Following study selection, data extraction was independently performed by R.M. and G.P. using a structured Microsoft Excel form. Extracted data included study characteristics (authors, year, country, design), participant demographics (sample size, mean age, sex distribution, cognitive status), intervention details, and outcome measures. For each cognitive and motor outcome, we collected the type of data reported (e.g., statistical significance, direction of effect, percentage improvement, or mean differences), as stated in the original articles.

### Risk of bias assessment

To ensure methodological rigor, risk of bias was assessed using the Cochrane Risk of Bias 2 (RoB 2)^57^ tool for RCTs and the ROBINS-I tool for non-randomized studies^58^. RoB 2 evaluates five domains: randomization, deviations from intended interventions, missing data, outcome measurement, and selection of reported results. ROBINS-I assesses confounding, participant selection, classification of interventions, deviations, missing data, outcome measurement, and reporting. The risk of bias for each included study was independently assessed by two reviewers (R.M. and G.P.), with disagreements resolved through discussion and, if needed, adjudicated by a third reviewer (M.G.M.).

### Data synthesis

Given the substantial heterogeneity in study designs, interventions, and outcome measures, a meta-analysis was not feasible. Consequently, a structured narrative synthesis was conducted in accordance with the SWiM guidelines to ensure transparency and consistency. Studies were grouped by neurological condition to facilitate clinically meaningful comparisons of exergaming effects. Outcomes were categorized into three domains: cognitive, motor, and adherence. Priority was given to RCTs when formulating conclusions, while quasi-experimental and feasibility studies were included to contextualize and enrich the interpretation of findings. Summary measures extracted from the included studies encompassed statistical significance (*p* values), direction of effect, and percentage improvement, where available. The synthesis was presented through descriptive summaries, comparative tables, and graphical visualizations, structured according to outcome domains. Heterogeneity was explored qualitatively, taking into account differences in interventions, settings, and outcome measures across studies.

The overall certainty of evidence for each outcome domain (cognitive, motor, and adherence) was assessed using the GRADE approach, which considers risk of bias, inconsistency, indirectness, imprecision, and publication bias. Although no formal statistical methods (e.g., funnel plots) were applied, potential publication bias was qualitatively evaluated based on the predominance of studies reporting positive results and the lack of pre-registered protocols or unpublished data. The strength of the body of evidence was rated as high, moderate, low, or very low. This evaluation was conducted independently by two reviewers (R.M. and G.P.), with discrepancies resolved by a third reviewer (M.G.M.).

## Supplementary information

Supplementary Information

Supplementary Information

Supplementary Information

## Data Availability

All data supporting the findings of this study are available within the manuscript. In addition, the underlying datasets can be accessed in the Supplementary Data 1, 2, Supplementary Table 2, Supplementary File S5, as well as in Figs. 1–3.

## References

[1] Package of interventions for rehabilitation: module 3: neurological conditions. https://www.who.int/publications/i/item/9789240071131 (2023).

[2] dos Santos, P. C. R., Orcioli-Silva, D., Vuillerme, N. & Barbieri, F. A. in *Locomotion and Posture in Older Adults: The Role of Aging and Movement Disorders* (eds Barbieri, F. A., Vitório, R. & dos Santos, P. C. R.) 207–232 (Springer Nature Switzerland, 2024).

[3] Herren, S. et al. Exergame (ExerG)-based physical-cognitive training for rehabilitation in adults with motor and balance impairments: usability study. *JMIR Serious Games* **13**, e66515 (2025). 39951650 10.2196/66515 PMC11844876

[4] Abd-Alrazaq, A. et al. The effectiveness of serious games in improving memory among older adults with cognitive impairment: systematic review and meta-analysis. *JMIR Serious Games* **10**, e35202 (2022). 35943792 10.2196/35202 PMC9399845

[5] Perez, F. M. P. et al. Decoding user experience in exergames: A systematic scoping review of assessment methods. *MethodsX* **14**, 103054 (2025). 39866200 10.1016/j.mex.2024.103054 PMC11764369

[6] Vacca, R. A. et al. Serious games in the new era of digital-health interventions: s narrative review of their therapeutic applications to manage neurobehavior in neurodevelopmental disorders. *Neurosci. Biobehav. Rev.* **149**, 105156 (2023). 37019246 10.1016/j.neubiorev.2023.105156

[7] Chan, J. Y. C., Liu, J., Chan, A. T. C. & Tsoi, K. K. F. Exergaming and cognitive functions in people with mild cognitive impairment and dementia: a meta-analysis. *npj Digit. Med.* **7**, 154 (2024). 38879695 10.1038/s41746-024-01142-4 PMC11180097

[8] Abd-alrazaq, A. et al. The performance of serious games for enhancing attention in cognitively impaired older adults. *npj Digit. Med.* **6**, 122 (2023). 37422507 10.1038/s41746-023-00863-2 PMC10329640

[9] Gonçalves, A. et al. The benefits of custom exergames for fitness, balance, and health-related quality of life: a randomized controlled trial with community-dwelling older adults. *Games Health J.* **10**, 245–253 (2021). 34370609 10.1089/g4h.2020.0092

[10] Born, F., Abramowski, S. & Masuch, M. Exergaming in VR: the impact of immersive embodiment on motivation, performance, and perceived exertion. In *2019 11th International Conference on Virtual Worlds and Games for Serious Applications. VS-Games* 1–8 (IEEE, 2019).

[11] Buele, J., Avilés-Castillo, F., Del-Valle-Soto, C., Varela-Aldás, J. & Palacios-Navarro, G. Effects of a dual intervention (motor and virtual reality-based cognitive) on cognition in patients with mild cognitive impairment: a single-blind, randomized controlled trial. *J. Neuroeng. Rehabil.* **21**, 130 (2024). 39090664 10.1186/s12984-024-01422-w PMC11293003

[12] Buele, J., Avilés-Castillo, F., Del-Valle-Soto, C., Varela-Aldás, J. & Palacios-Navarro, G. Correction: Effects of a dual intervention (motor and virtual reality-based cognitive) on cognition in patients with mild cognitive impairment: a single-blind, randomized controlled trial. *J. Neuroeng. Rehabil.* **21**, 149 (2024). 39090664 10.1186/s12984-024-01422-w PMC11293003

[13] Chien, T.-Y., Chern, J.-S., Wang, S.-P. & Yang, Y. Effects of multitask training on cognition and motor control in people with schizophrenia spectrum disorders. *PLoS ONE* **17**, e0264745 (2022). 35771832 10.1371/journal.pone.0264745 PMC9246115

[14] Wallin, A. et al. Balance exercise interventions in Parkinson’s disease: a systematic mapping review of components, progression, and intensity. *Parkinsonism Relat. Disord*. **133**, 107310 (2025). 10.1016/j.parkreldis.2025.107310 39915205

[15] Janhunen, M. et al. Effectiveness of exergame intervention on walking in older adults: a systematic review and meta-analysis of randomized controlled trials. *Phys. Ther.* **101**, pzab152 (2021). 34160022 10.1093/ptj/pzab152 PMC8459884

[16] Staiano, A. E. & Calvert, S. L. The promise of exergames as tools to measure physical health. *Entertain. Comput.* **2**, 17–21 (2011). 23378860 10.1016/j.entcom.2011.03.008 PMC3562354

[17] Warburton, D. E. R. et al. The health benefits of interactive video game exercise. *Appl. Physiol. Nutr. Metab. Physiol. Appl. Nutr. Metab.* **32**, 655–663 (2007). 10.1139/H07-038 17622279

[18] Zeng, N., Pope, Z., Lee, J. E. & Gao, Z. A systematic review of active video games on rehabilitative outcomes among older patients. *J. Sport Health Sci.* **6**, 33–43 (2017). 30356538 10.1016/j.jshs.2016.12.002 PMC6188917

[19] Schatton, C. et al. Individualized exergame training improves postural control in advanced degenerative spinocerebellar ataxia: a rater-blinded, intra-individually controlled trial. *Parkinsonism Relat. Disord.* **39**, 80–84 (2017). 28365204 10.1016/j.parkreldis.2017.03.016

[20] Glavare, M., Stålnacke, B.-M., Häger, C. K. & Löfgren, M. Virtual reality exercises in an interdisciplinary rehabilitation programme for persons with chronic neck pain: a feasibility study. *J. Rehabil. Med. Clin. Commun.* **4**, 1000067 (2021). 34527201 10.2340/20030711-1000067 PMC8404524

[21] Maranesi, E. et al. The effect of non-immersive virtual reality exergames versus traditional physiotherapy in parkinson’s disease older patients: preliminary results from a randomized-controlled trial. *Int. J. Environ. Res. Public. Health* **19**, 14818 (2022). 36429537 10.3390/ijerph192214818 PMC9690935

[22] Saeed, A. et al. A difficulty based comparison of novel exergame balance training for cognitive functions in adults with mild cognitive impairment: a randomized trial. *Ment. Health Phys. Act.* **27**, 100637 (2024).

[23] Manser, P. & de Bruin, E. D. Brain-IT’: exergame training with biofeedback breathing in neurocognitive disorders. *Alzheimers Dement.* **20**, 4747–4764 (2024). 38809948 10.1002/alz.13913 PMC11247687

[24] Swinnen, N. et al. The efficacy of exergaming in people with major neurocognitive disorder residing in long-term care facilities: a pilot randomized controlled trial. *Alzheimers Res. Ther.* **13**, 70 (2021). 33785077 10.1186/s13195-021-00806-7 PMC8008333

[25] Li, A. et al. Evaluating the clinical efficacy of an exergame-based training program for enhancing physical and cognitive functions in older adults with mild cognitive impairment and dementia residing in rural long-term care facilities: randomized controlled trial. *J. Med. Internet Res.* **27**, e69109 (2025). 39969990 10.2196/69109 PMC11888014

[26] Maier, M., Ballester, B. R., Leiva Bañuelos, N., Duarte Oller, E. & Verschure, P. F. M. J. Adaptive conjunctive cognitive training (ACCT) in virtual reality for chronic stroke patients: a randomized controlled pilot trial. *J. Neuroeng. Rehabil.* **17**, 42 (2020). 32143674 10.1186/s12984-020-0652-3 PMC7059385

[27] Kannan, L., Vora, J., Bhatt, T. & Hughes, S. L. Cognitive-motor exergaming for reducing fall risk in people with chronic stroke: a randomized controlled trial. *Neurorehabilitation* **44**, 493–510 (2019). 31256084 10.3233/NRE-182683

[28] Salisbury, D. L., Pituch, K. A. & Yu, F. The effects of exergame telerehabilitation in persons with subjective cognitive decline. *Gerontologist* **64**, gnae028 (2024). 38486359 10.1093/geront/gnae028 PMC11138364

[29] Huber, S. K., Held, J. P. O., de Bruin, E. D. & Knols, R. H. Personalized motor-cognitive exergame training in chronic stroke patients—a feasibility study. *Front. Aging Neurosci*. **13**, 730801 (2021). 10.3389/fnagi.2021.730801 PMC8565485 34744688

[30] Manser, P., Poikonen, H. & de Bruin, E. D. Feasibility, usability, and acceptance of “Brain-IT”—a newly developed exergame-based training concept for the secondary prevention of mild neurocognitive disorder: a pilot randomized controlled trial. *Front. Aging Neurosci*. **15**, 1163388 (2023). 10.3389/fnagi.2023.1163388 PMC10557950 37810620

[31] Werner, C. et al. Time course of changes in motor-cognitive exergame performances during task-specific training in patients with dementia: identification and predictors of early training response. *J. Neuroeng. Rehabil.* **15**, 100 (2018). 30409202 10.1186/s12984-018-0433-4 PMC6225709

[32] Yun, S. J., Hyun, S. E., Oh, B.-M. & Seo, H. G. Fully immersive virtual reality exergames with dual-task components for patients with Parkinson’s disease: a feasibility study. *J. Neuroeng. Rehabil.* **20**, 92 (2023). 37464349 10.1186/s12984-023-01215-7 PMC10355082

[33] Kannan, L. et al. Gaming-based tele-exercise program to improve physical function in frail older adults: feasibility randomized controlled trial. *J. Med. Internet Res.* **26**, e56810 (2024). 39602215 10.2196/56810 PMC11635319

[34] Schättin, A. et al. Design and evaluation of user-centered exergames for patients with multiple sclerosis: multilevel usability and feasibility studies. *JMIR Serious Games* **9**, e22826 (2021). 33960956 10.2196/22826 PMC8140386

[35] Marston, H. R. & Smith, S. T. Interactive videogame technologies to support independence in the elderly: a narrative review. *Games Health J.* **1**, 139–152 (2012). 26193189 10.1089/g4h.2011.0008

[36] Maldonado-Díaz, M. et al. Teleneurorehabilitation program (virtual reality) for patients with balance disorders: descriptive study. *BMC Sports Sci. Med. Rehabil.* **13**, 83 (2021). 34340687 10.1186/s13102-021-00314-z PMC8330090

[37] Feng, P., Wang, B., Liu, D. L. & Yu, Q. Machine learning-based integration of remotely-sensed drought factors can improve the estimation of agricultural drought in South-Eastern Australia. *Agric. Syst.* **173**, 303–316 (2019).

[38] Calafiore, D. et al. Efficacy of virtual reality and exergaming in improving balance in patients with multiple sclerosis: a systematic review and meta-analysis. *Front. Neurol.* **12**, 773459 (2021). 34956054 10.3389/fneur.2021.773459 PMC8702427

[39] Saragih, I. D., Gervais, W., Lamora, J.-P., Batcho, C. S. & Everard, G. Effect of serious games over conventional therapy in the rehabilitation of people with multiple sclerosis - a systematic review and meta-analysis. *Disabil. Rehabil*. **47**, 3224–3244 (2024). 10.1080/09638288.2024.2415328 39421950

[40] Stanmore, E., Stubbs, B., Vancampfort, D., de Bruin, E. D. & Firth, J. The effect of active video games on cognitive functioning in clinical and non-clinical populations: a meta-analysis of randomized controlled trials. *Neurosci. Biobehav. Rev.* **78**, 34–43 (2017). 28442405 10.1016/j.neubiorev.2017.04.011

[41] Sturnieks, D. L. et al. Exergame and cognitive training for preventing falls in community-dwelling older people: a randomized controlled trial. *Nat. Med.* **30**, 98–105 (2024). 38228913 10.1038/s41591-023-02739-0

[42] Huber, S. K., Manser, P. & de Bruin, E. D. PEMOCS: theory derivation of a concept for PErsonalized MOtor-Cognitive exergame training in chronic Stroke—a methodological paper with an application example. *Front. Sports Act. Living* **6**, 1397949 (2024). 10.3389/fspor.2024.1397949 PMC11194322 38915297

[43] Hortobágyi, T. et al. The impact of aerobic and resistance training intensity on markers of neuroplasticity in health and disease. *Ageing Res. Rev.* **80**, 101698 (2022). 35853549 10.1016/j.arr.2022.101698

[44] Seinsche, J. et al. A newly developed exergame-based telerehabilitation system for older adults: usability and technology acceptance study. *JMIR Hum. Factors* **10**, e48845 (2023). 38060283 10.2196/48845 PMC10739244

[45] Dufresne, F., Dubosc, C., Lefrou, T., Gorisse, G. & Christmann, O. Am I (not) a ghost? Leveraging affordances to study the impact of avatar/interaction coherence on embodiment and plausibility in virtual reality. *IEEE Trans. Vis. Comput. Graph*. **31**, 2611–2621 (2025). 10.1109/TVCG.2025.3549136 40053655

[46] Do, T. D., Benjamin, J., Protko, C. I. & McMahan, R. P. Cultural reflections in virtual reality: the effects of user ethnicity in Avatar matching experiences on sense of embodiment. *IEEE Trans. Vis. Comput. Graph.* **30**, 7408–7418 (2024). 39250390 10.1109/TVCG.2024.3456196

[47] Impellizzeri, F. et al. Does cybersickness affect virtual reality training using the Computer Assisted Rehabilitation Environment (CAREN)? Preliminary results from a case-control study in Parkinson’s disease. *Physiother. Theory Pract.* **38**, 2603–2611 (2022). 34365911 10.1080/09593985.2021.1964117

[48] O’Neil, O. et al. Virtual reality for neurorehabilitation: insights from 3 European clinics. *PM R* **10**, S198–S206 (2018). 30121365 10.1016/j.pmrj.2018.08.375

[49] Perez-Marcos, D., Bieler-Aeschlimann, M. & Serino, A. Virtual reality as a vehicle to empower motor-cognitive neurorehabilitation. *Front. Psychol*. **9**, 2120 (2018). 10.3389/fpsyg.2018.02120 PMC6224455 30450069

[50] Eggenberger, P., Wolf, M., Schumann, M. & de Bruin, E. D. Exergame and balance training modulate prefrontal brain activity during walking and enhance executive function in older adults. *Front. Aging Neurosci.* **8**, 66 (2016). 27148041 10.3389/fnagi.2016.00066 PMC4828439

[51] Eggenberger, P., Schumacher, V., Angst, M., Theill, N. & de Bruin, E. D. Does multicomponent physical exercise with simultaneous cognitive training boost cognitive performance in older adults? A 6-month randomized controlled trial with a 1-year follow-up. *Clin. Interv. Aging* **10**, 1335–1349 (2015). 26316729 10.2147/CIA.S87732 PMC4544626

[52] Seidler, R. D. et al. Motor control and aging: links to age-related brain structural, functional, and biochemical effects. *Neurosci. Biobehav. Rev.* **34**, 721–733 (2010). 19850077 10.1016/j.neubiorev.2009.10.005 PMC2838968

[53] Nicastri, C. M. et al. BDNF mediates improvement in cognitive performance after computerized cognitive training in healthy older adults. *Alzheimers Dement.* **8**, e12337 (2022). 10.1002/trc2.12337 PMC9428279 36089933

[54] Page, M. J. et al. The PRISMA 2020 statement: an updated guideline for reporting systematic reviews. *BMJ* **372**, n71 (2021). 33782057 10.1136/bmj.n71 PMC8005924

[55] Higgins, J. P., Savović, J., Page, M. J., Elbers, R. G. & Sterne, J. A. in *Cochrane Handbook for Systematic Reviews of Interventions* 205–228 (Wiley, 2019).

[56] Campbell, M. et al. Synthesis without meta-analysis (SWiM) in systematic reviews: reporting guideline. *BMJ* **368**, l6890 (2020). 31948937 10.1136/bmj.l6890 PMC7190266

[57] Sterne, J. A. C. et al. RoB 2: a revised tool for assessing risk of bias in randomised trials. *BMJ* **366**, l4898 (2019). 31462531 10.1136/bmj.l4898

[58] Sterne, J. A. et al. ROBINS-I: a tool for assessing risk of bias in non-randomised studies of interventions. *BMJ* **355**, i4919 (2016). 27733354 10.1136/bmj.i4919 PMC5062054

