# Supplementary material for: The overlooked role of exergames in cognitive-motor neurorehabilitation: a systematic review
Source: NPJ Digit Med. 2025 Jul 9;8:419. doi: 10.1038/s41746-025-01843-4 (PMC12241605; doi:10.1038/s41746-025-01843-4)
Supplement: Supplementary file 1 — Supplementary Information [file 41746_2025_1843_MOESM1_ESM.pdf]

Supplementary Data 1. Revised database search strategies used in the systematic review, including the updated strings for each database after removing temporal filters and re-running the searches.

Supplementary Data 2. Risk of bias assessment of the included studies using the RoB 2 tool for randomized controlled trials and the ROBINS I tool for non-randomized studies.

Supplementary table 1. In accordance with PRISMA guidelines (Item 8), to ensure transparency and reproducibility.

Database	MESH Terms/Keywords Strings
Pubmed	1. ("motor function" OR "motor learning" OR "motor rehabilitation") AND ("cognitive function" OR "executive function" OR "cognition" OR "dual task") AND ("exergame");<br>2. ("exergamers"[All Fields] OR "exergaming"[MeSH Terms] OR "exergaming"[All Fields] OR "exergame"[All Fields] OR "exergames"[All Fields]) AND (("motor"[All Fields] OR "motor s"[All Fields]) AND ("functional"[All Fields] OR "functioning"[All Fields] OR "functions"[All Fields])) AND ("cognition"[MeSH Terms] OR "cognition"[All Fields] OR ("cognitive"[All Fields] AND "function"[All Fields]) OR "cognitive function"[All Fields]).<br>3. "Dual"[All Fields] AND "Task"[All Fields] AND ("exergamers"[All Fields] OR "exergaming"[MeSH Terms] OR "exergaming"[All Fields] OR "exergame"[All Fields] OR "exergames"[All Fields]);<br>4. ("motor function" OR "motor control" OR "motor learning" OR "motor rehabilitation") AND ("cognitive function" OR "cognition" OR "dual task") AND ("exergame") AND ("neuroplasticity" OR "brain connectivity");<br>5. ("Virtual Reality"[MeSH Terms] OR "Virtual Reality"[Title/Abstract]) AND ("Video Games"[MeSH Terms] OR "Serious Games"[MeSH Terms] OR "Games"[Title/Abstract]) AND ("Rehabilitation"[MeSH Terms] OR "Cognitive Therapy"[MeSH Terms] OR "Physical Therapy Modalities"[MeSH Terms] OR rehabilitation[Title/Abstract]) AND ("Motor Skills"[MeSH Terms] OR "Cognition"[MeSH Terms] OR "Dual Task"[Title/Abstract])<br>6. ("Telerehabilitation"[MeSH Terms] OR "Remote Consultation"[MeSH Terms] OR telerehabilitation[Title/Abstract]) AND ("Virtual Reality"[MeSH Terms] OR "Virtual Reality Exposure Therapy"[MeSH Terms] OR "Virtual Reality"[Title/Abstract]) AND ("Video Games"[MeSH Terms] OR "Exergames"[MeSH Terms] OR "Computer Simulation"[MeSH Terms] OR games[Title/Abstract]) AND

	("Motor Activity"[MeSH Terms] OR "Motor Skills"[MeSH Terms] OR "Movement"[MeSH Terms] OR "motor function"[Title/Abstract]) AND ("Cognition"[MeSH Terms] OR "Cognitive Dysfunction"[MeSH Terms] OR "Cognition Disorders"[MeSH Terms] OR "cognitive function"[Title/Abstract]) AND ("Rehabilitation"[MeSH Terms] OR rehabilitation[Title/Abstract])
Scopus	1. ("motor function" OR "motor learning" OR "motor rehabilitation") AND ("cognitive function" OR "executive function" OR "cognition" OR "dual task") AND ("exergame");<br>2. ("exergamers"[All Fields] OR "exergaming"[MeSH Terms] OR "exergaming"[All Fields] OR "exergame"[All Fields] OR "exergames"[All Fields]) AND (("motor"[All Fields] OR "motor s"[All Fields]) AND ("functional"[All Fields] OR "functioning"[All Fields] OR "functions"[All Fields])) AND ("cognition"[MeSH Terms] OR "cognition"[All Fields] OR ("cognitive"[All Fields] AND "function"[All Fields]) OR "cognitive function"[All Fields]).<br>3. "Dual"[All Fields] AND "Task"[All Fields] AND ("exergamers"[All Fields] OR "exergaming"[MeSH Terms] OR "exergaming"[All Fields] OR "exergame"[All Fields] OR "exergames"[All Fields]);<br>4. ("motor function" OR "motor control" OR "motor learning" OR "motor rehabilitation") AND ("cognitive function" OR "cognition" OR "dual task") AND ("exergame") AND ("neuroplasticity" OR "brain connectivity");<br>5. ("Virtual Reality"[MeSH Terms] OR "Virtual Reality"[Title/Abstract]) AND ("Video Games"[MeSH Terms] OR "Serious Games"[MeSH Terms] OR "Games"[Title/Abstract]) AND ("Rehabilitation"[MeSH Terms] OR "Cognitive Therapy"[MeSH Terms] OR "Physical Therapy Modalities"[MeSH Terms] OR rehabilitation[Title/Abstract]) AND ("Motor Skills"[MeSH Terms] OR "Cognition"[MeSH Terms] OR "Dual Task"[Title/Abstract])<br>6. ("Telerehabilitation"[MeSH Terms] OR "Remote Consultation"[MeSH Terms] OR telerehabilitation[Title/Abstract]) AND ("Virtual Reality"[MeSH Terms] OR "Virtual Reality Exposure Therapy"[MeSH Terms] OR "Virtual Reality"[Title/Abstract]) AND ("Video Games"[MeSH Terms] OR "Exergames"[MeSH Terms] OR "Computer Simulation"[MeSH Terms] OR games[Title/Abstract]) AND ("Motor Activity"[MeSH Terms] OR "Motor Skills"[MeSH Terms] OR "Movement"[MeSH Terms] OR "motor function"[Title/Abstract]) AND ("Cognition"[MeSH Terms] OR "Cognitive Dysfunction"[MeSH Terms] OR "Cognition Disorders"[MeSH Terms] OR "cognitive function"[Title/Abstract]) AND ("Rehabilitation"[MeSH Terms] OR rehabilitation[Title/Abstract])

Web Of Science	1. ("motor function" OR "motor learning" OR "motor rehabilitation") AND ("cognitive function" OR "executive function" OR "cognition" OR "dual task") AND ("exergame");<br>2. ("exergamers"[All Fields] OR "exergaming"[MeSH Terms] OR "exergaming"[All Fields] OR "exergame"[All Fields] OR "exergames"[All Fields]) AND (("motor"[All Fields] OR "motor s"[All Fields])

	AND ("functional"[All Fields] OR "functioning"[All Fields] OR "functions"[All Fields])) AND ("cognition"[MeSH Terms] OR "cognition"[All Fields] OR ("cognitive"[All Fields] AND "function"[All Fields]) OR "cognitive function"[All Fields]).<br>3. "Dual"[All Fields] AND "Task"[All Fields] AND ("exergamers"[All Fields] OR "exergaming"[MeSH Terms] OR "exergaming"[All Fields] OR "exergame"[All Fields] OR "exergames"[All Fields]);<br>4. ("motor function" OR "motor control" OR "motor learning" OR "motor rehabilitation") AND ("cognitive function" OR "cognition" OR "dual task") AND ("exergame") AND ("neuroplasticity" OR "brain connectivity");<br>5. ("Virtual Reality"[MeSH Terms] OR "Virtual Reality"[Title/Abstract]) AND ("Video Games"[MeSH Terms] OR "Serious Games"[MeSH Terms] OR "Games"[Title/Abstract]) AND ("Rehabilitation"[MeSH Terms] OR "Cognitive Therapy"[MeSH Terms] OR "Physical Therapy Modalities"[MeSH Terms] OR rehabilitation[Title/Abstract]) AND ("Motor Skills"[MeSH Terms] OR "Cognition"[MeSH Terms] OR "Dual Task"[Title/Abstract])<br>6. ("Telerehabilitation"[MeSH Terms] OR "Remote Consultation"[MeSH Terms] OR telerehabilitation[Title/Abstract]) AND ("Virtual Reality"[MeSH Terms] OR "Virtual Reality Exposure Therapy"[MeSH Terms] OR "Virtual Reality"[Title/Abstract]) AND ("Video Games"[MeSH Terms] OR "Exergames"[MeSH Terms] OR "Computer Simulation"[MeSH Terms] OR games[Title/Abstract]) AND ("Motor Activity"[MeSH Terms] OR "Motor Skills"[MeSH Terms] OR "Movement"[MeSH Terms] OR "motor function"[Title/Abstract]) AND ("Cognition"[MeSH Terms] OR "Cognitive Dysfunction"[MeSH Terms] OR "Cognition Disorders"[MeSH Terms] OR "cognitive function"[Title/Abstract]) AND ("Rehabilitation"[MeSH Terms] OR rehabilitation[Title/Abstract])
Embase	1. ("motor function" OR "motor learning" OR "motor rehabilitation") AND ("cognitive function" OR "executive function" OR "cognition" OR "dual task") AND ("exergame");<br>2. ("exergamers"[All Fields] OR "exergaming"[MeSH Terms] OR "exergaming"[All Fields] OR "exergame"[All Fields] OR "exergames"[All Fields]) AND (("motor"[All Fields] OR "motor s"[All Fields]) AND ("functional"[All Fields] OR "functioning"[All Fields] OR "functions"[All Fields])) AND ("cognition"[MeSH Terms] OR "cognition"[All Fields] OR ("cognitive"[All Fields] AND "function"[All Fields]) OR "cognitive function"[All Fields]).<br>3. "Dual"[All Fields] AND "Task"[All Fields] AND ("exergamers"[All Fields] OR "exergaming"[MeSH Terms] OR "exergaming"[All Fields] OR "exergame"[All Fields] OR "exergames"[All Fields]);

4. ("motor function" OR "motor control" OR "motor learning" OR "motor rehabilitation") AND ("cognitive function" OR "cognition" OR "dual task") AND ("exergame") AND ("neuroplasticity" OR "brain connectivity");
5. ("Virtual Reality"[MeSH Terms] OR "Virtual Reality"[Title/Abstract]) AND ("Video Games"[MeSH Terms] OR "Serious Games"[MeSH Terms] OR "Games"[Title/Abstract]) AND ("Rehabilitation"[MeSH Terms] OR "Cognitive Therapy"[MeSH Terms] OR "Physical Therapy Modalities"[MeSH Terms] OR rehabilitation[Title/Abstract]) AND ("Motor Skills"[MeSH Terms] OR "Cognition"[MeSH Terms] OR "Dual Task"[Title/Abstract])
6. ("Telerehabilitation"[MeSH Terms] OR "Remote Consultation"[MeSH Terms] OR telerehabilitation[Title/Abstract]) AND ("Virtual Reality"[MeSH Terms] OR "Virtual Reality Exposure Therapy"[MeSH Terms] OR "Virtual Reality"[Title/Abstract]) AND ("Video Games"[MeSH Terms] OR "Exergames"[MeSH Terms] OR "Computer Simulation"[MeSH Terms] OR games[Title/Abstract]) AND ("Motor Activity"[MeSH Terms] OR "Motor Skills"[MeSH Terms] OR "Movement"[MeSH Terms] OR "motor function"[Title/Abstract]) AND ("Cognition"[MeSH Terms] OR "Cognitive Dysfunction"[MeSH Terms] OR "Cognition Disorders"[MeSH Terms] OR "cognitive function"[Title/Abstract]) AND ("Rehabilitation"[MeSH Terms] OR rehabilitation[Title/Abstract])

Supplementary table 2. Summary of GRADE assessment for cognitive, motor, and feasibility/usability outcomes. This table provides an overview of the certainty of evidence for each outcome category, based on risk of bias, inconsistency, indirectness, imprecision, and potential publication bias, following the GRADE framework. Overall certainty was rated as moderate for cognitive and motor outcomes and low for feasibility/usability, primarily due to small sample sizes, heterogeneous protocols, and variability in outcome measurement.

Outcome	Study Design	Risk of Bias	Inconsistency	Indirectness	Imprecision	Publication Bias	Overall Certainty
Cognitive outcomes	RCTs, non-RCTs	No serious concerns	Minor variability in cognitive measures	No serious concerns	Some studies with small samples and wide CIs	Unlikely	Moderate
Motor outcomes	RCTs, non-RCTs	No serious concerns	Moderate heterogeneity in motor tests used	No serious concerns	Sample variability	Unlikely	Moderate
Feasibility/usability	RCTs, non-RCTs	Low to moderate	Low: consistent results across studies	No serious concerns	Qualitative data, small samples	Unlikely	Low

Supplementary table 3. PRISMA checklist.

PRISMA 2020 Checklist

Section and Topic	Item #	Checklist item	Location where item is reported
**TITLE**			
Title	1	Identify the report as a systematic review.	1
**ABSTRACT**			
Abstract	2	See the PRISMA 2020 for Abstracts checklist.	1-2
**INTRODUCTION**			
Rationale	3	Describe the rationale for the review in the context of existing knowledge.	2-3
Objectives	4	Provide an explicit statement of the objective(s) or question(s) the review addresses.	4
**METHODS**			
Eligibility criteria	5	Specify the inclusion and exclusion criteria for the review and how studies were grouped for the syntheses.	4-5
Information sources	6	Specify all databases, registers, websites, organisations, reference lists and other sources searched or consulted to identify studies. Specify the date when each source was last searched or consulted.	5-6
Search strategy	7	Present the full search strategies for all databases, registers and websites, including any filters and limits used.	5-7
Selection process	8	Specify the methods used to decide whether a study met the inclusion criteria of the review, including how many reviewers screened each record and each report retrieved, whether they worked independently, and if applicable, details of automation tools used in the process.	7-8
Data collection process	9	Specify the methods used to collect data from reports, including how many reviewers collected data from each report, whether they worked independently, any processes for obtaining or confirming data from study investigators, and if applicable, details of automation tools used in the process.	6-8
Data items	10a	List and define all outcomes for which data were sought. Specify whether all results that were compatible with each outcome domain in each study were sought (e.g. for all measures, time points, analyses), and if not, the methods used to decide which results to collect.	8-9
	10b	List and define all other variables for which data were sought (e.g. participant and intervention characteristics, funding sources). Describe any assumptions made about any missing or unclear information.	6
Study risk of bias assessment	11	Specify the methods used to assess risk of bias in the included studies, including details of the tool(s) used, how many reviewers assessed each study and whether they worked independently, and if applicable, details of automation tools used in the process.	9
Effect measures	12	Specify for each outcome the effect measure(s) (e.g. risk ratio, mean difference) used in the synthesis or presentation of results.	7
Synthesis methods	13a	Describe the processes used to decide which studies were eligible for each synthesis (e.g. tabulating the study intervention characteristics and comparing against the planned groups for each synthesis (item #5)).	4-5
	13b	Describe any methods required to prepare the data for presentation or synthesis, such as handling of missing summary statistics, or data conversions.	4
	13c	Describe any methods used to tabulate or visually display results of individual studies and syntheses.	8
	13d	Describe any methods used to synthesize results and provide a rationale for the choice(s). If meta-analysis was performed, describe the model(s), method(s) to identify the presence and extent of statistical heterogeneity, and software package(s) used.	7-8
	13e	Describe any methods used to explore possible causes of heterogeneity among study results (e.g. subgroup analysis, meta-regression).	7-8
	13f	Describe any sensitivity analyses conducted to assess robustness of the synthesized results.	7-8
Reporting bias assessment	14	Describe any methods used to assess risk of bias due to missing results in a synthesis (arising from reporting biases).	9
Certainty assessment	15	Describe any methods used to assess certainty (or confidence) in the body of evidence for an outcome.	9-10

PRISMA 2020 Checklist

Section and Topic	Item #	Checklist item	Location where item is reported
**RESULTS**			
Study selection	16a	Describe the results of the search and selection process, from the number of records identified in the search to the number of studies included in the review, ideally using a flow diagram.	8-9
	16b	Cite studies that might appear to meet the inclusion criteria, but which were excluded, and explain why they were excluded.	8
Study characteristics	17	Cite each included study and present its characteristics.	10-13
Risk of bias in studies	18	Present assessments of risk of bias for each included study.	9
Results of individual studies	19	For all outcomes, present, for each study: (a) summary statistics for each group (where appropriate) and (b) an effect estimate and its precision (e.g. confidence/credible interval), ideally using structured tables or plots.	8<br>Table 2
Results of syntheses	20a	For each synthesis, briefly summarise the characteristics and risk of bias among contributing studies.	9-13
	20b	Present results of all statistical syntheses conducted. If meta-analysis was done, present for each the summary estimate and its precision (e.g. confidence/credible interval) and measures of statistical heterogeneity. If comparing groups, describe the direction of the effect.	8-13<br>Table 2
	20c	Present results of all investigations of possible causes of heterogeneity among study results.	10
	20d	Present results of all sensitivity analyses conducted to assess the robustness of the synthesized results.	10-13
Reporting biases	21	Present assessments of risk of bias due to missing results (arising from reporting biases) for each synthesis assessed.	9-10
Certainty of evidence	22	Present assessments of certainty (or confidence) in the body of evidence for each outcome assessed.	9-10
**DISCUSSION**			
Discussion	23a	Provide a general interpretation of the results in the context of other evidence.	13-15
	23b	Discuss any limitations of the evidence included in the review.	17-19
	23c	Discuss any limitations of the review processes used.	21-22
	23d	Discuss implications of the results for practice, policy, and future research.	19-20
**OTHER INFORMATION**			
Registration and protocol	24a	Provide registration information for the review, including register name and registration number, or state that the review was not registered.	2
	24b	Indicate where the review protocol can be accessed, or state that a protocol was not prepared.	2
	24c	Describe and explain any amendments to information provided at registration or in the protocol.	N/A
Support	25	Describe sources of financial or non-financial support for the review, and the role of the funders or sponsors in the review.	28
Competing interests	26	Declare any competing interests of review authors.	29
Availability of data, code and other materials	27	Report which of the following are publicly available and where they can be found: template data collection forms; data extracted from included studies; data used for all analyses; analytic code; any other materials used in the review.	29

From: Page MJ, McKenzie JE, Bossuyt PM, Boutron I, Hoffmann TC, Mulrow CD, et al. The PRISMA 2020 statement: an updated guideline for reporting systematic reviews. BMJ 2021;372:n71. doi: 10.1136/bmj.n71. This work is licensed under CC BY 4.0. To view a copy of this license, visit https://creativecommons.org/licenses/by/4.0/

Supplementary table 4. Studies included in the analysis.

TYPE OF DISEASE	AUTHOR INFORMATION	OUTCOME MEASURES & EFFICACY	MAIN FINDINGS
MCI	Saeed et al., 2024	Montreal Cognitive Assessment (MoCA), Trail Making Test, Stroop Test, ADAS Word List, Digit Span, Counting Backward Test	Significant improvement in global cognition, inhibitory control, working memory, and attention in all exergame groups compared to control; Higher cognitive challenge led to greater clinical improvement
	Li et al., 2025	Sit-and-reach test, shoulder flexibility test, trunk rotation flexibility, range of motion, motor coordination (figure-of-8 walk test), hand dexterity test, cognitive tests (CASI, MMSE, MoCA)	Significant improvements in flexibility, joint mobility, motor coordination, hand dexterity, and cognitive function in the exergame group compared to controls
	Manser & de Bruin, 2024	Quick Mild Cognitive Impairment screen (QMCI), Learning & Memory tests (WMS-IV-LM), Executive Function (Trail Making Test, Go/No-Go), Gait Analysis, HRV metrics	Significant improvement in global cognition (large effect size), immediate and delayed verbal recall; 55% of participants showed clinically relevant improvement
	Manser et al., 2023	System Usability Scale (SUS), Exergame Enjoyment Questionnaire (EEQ), Behavioral Regulation in Exercise Questionnaire (BREQ); Pre-post measurements for cognitive function, gait, and heart rate variability	Training was feasible and well-accepted; High adherence (85%), good usability (SUS=71.7), and increased motivation; Preliminary data suggest cognitive and physical benefits
	Swinnen et al., 2021	Improved outcomes in exergame group: gait speed (SPPB), cognitive function (MoCA), step reaction time (SRTT), reduced depression (CSDD)<br>No significant differences in neuropsychiatric symptoms (NPI), quality of life (DQoL), and ADL<br>Attendance: Exergame group 82.9%, Control group 73.7%	Exergaming improved physical, cognitive, and mental health outcomes: gait speed, cognitive function, depression reduction.<br>No study-related adverse events reported

	Werner et al., 2018	Time required to complete Physiomat® tasks, Trail Making Test, Timed Up and Go, Dual-task cost (walking while counting)	Significant improvements in exergame performance within 3 weeks; Participants with lower initial performance and visuospatial/divided attention deficits showed the fastest improvements
	Salisbury et al., 2024	NIH Toolbox Cognitive Battery, 6-Minute Walk Test (6MWT), Shuttle Walk Test (SWT)	Significant improvement in fluid cognition within the exergame group; No significant between-group differences in cognition or aerobic fitness
Stroke	Kannan et al., 2019	Balance: Limits of Stability (LOS), Slip-Perturbation Test (SPT); Cognition: Letter-Number Sequencing (LNS)	Cognitive-motor exergaming improved volitional and reactive balance control under dual-task conditions, unlike conventional balance training
	Huber et al., 2021	Feasibility (adherence, compliance, motivation, satisfaction), Mobility (Timed-Up-and-Go, gait analysis), Cognition (TMT, MRT, TAP tests)	Feasible intervention with high adherence (95%) and motivation (77%); Significant improvement in TUG ($p=0.05$, $r=0.46$); Medium effect sizes for cognitive improvements (TMT, MRT)
	Maier et al., 2020	Neuropsychological test battery (attention, memory, executive function, spatial awareness); HAM-D; MoCA; MMSE; Barthel Index	Significant improvements in attention, spatial awareness, and general cognition in experimental group. Depression scores also improved. No significant changes in executive function.
PD	Yun et al., 2023	Improvement in BBS and Stroop color–word test ($p = 0.047$, $p = 0.003$)<br>No significant change in TUG, dual-task performance, or UPDRS	VR exergames led to improvement in executive function (Stroop test) and balance (BBS)<br>Participants demonstrated high satisfaction and a low rate of

	adverse events (only mild blurred vision reported)
